# Higher-Dimensional Automorphic Lie Algebras

**DOI:** 10.1007/s10208-016-9312-1

**Published:** 2016-04-11

**Authors:** Vincent Knibbeler, Sara Lombardo, Jan A. Sanders

**Affiliations:** 1 Department of Mathematics, Faculty of Sciences, Vrije Universiteit, De Boelelaan 1081a, 1081 HV Amsterdam, The Netherlands; 2 Department of Mathematics and Information Sciences, Northumbria University, Newcastle upon Tyne, NE1 8ST UK

**Keywords:** Automorphic Lie Algebras, Infinite-dimensional Lie algebras, Chevalley normal forms, 16Z05, 17B05, 17B65, 17B80

## Abstract

The paper presents the complete classification of Automorphic Lie Algebras based on ${{\mathfrak {sl}}}_{n}(\mathbb {C})$, where the symmetry group *G* is finite and acts on ${{\mathfrak {sl}}}_n(\mathbb {C})$ by inner automorphisms, ${{\mathfrak {sl}}}_n(\mathbb {C})$ has no trivial summands, and where the poles are in any of the exceptional *G*-orbits in $\overline{\mathbb {C}}$. A key feature of the classification is the study of the algebras in the context of *classical invariant theory*. This provides on the one hand a powerful tool from the computational point of view; on the other, it opens new questions from an algebraic perspective (e.g. structure theory), which suggest further applications of these algebras, beyond the context of integrable systems. In particular, the research shows that this class of Automorphic Lie Algebras associated with the $\mathbb {T}\mathbb {O}\mathbb {Y}$ groups (tetrahedral, octahedral and icosahedral groups) depend on the group through the automorphic functions only; thus, they are group independent as Lie algebras. This can be established by defining a *Chevalley normal form* for these algebras, generalising this classical notion to the case of Lie algebras over a polynomial ring.

## Introduction

An Automorphic Lie Algebra (ALiA in what follows) is the space of invariants

$$\begin{aligned} ({{\mathfrak {g}}}\otimes {\mathcal {M}}(\overline{\mathbb {C}}))^G_\Gamma \end{aligned}$$

obtained by imposing a finite group symmetry on a current algebra of Krichever–Novikov (KN) type [31] ${{\mathfrak {g}}}\otimes {\mathcal {M}}(\overline{\mathbb {C}})$ where ${{\mathfrak {g}}}$ is a complex Lie algebra, ${\mathcal {M}}(\overline{\mathbb {C}})$ the field of meromorphic functions on the Riemann sphere $\overline{\mathbb {C}}=\mathbb {C}\cup \{\infty \}$, *G* a subgroup of $\text {Aut}({{\mathfrak {g}}}\otimes {\mathcal {M}}(\overline{\mathbb {C}}))$ and where $\Gamma \subset \overline{\mathbb {C}}$ is a *G*-orbit, to which poles are confined. Since their introduction in [24] automorphic algebras have been extensively studied (see [25] and references therein, but also [3, 4]), ALiAs arose originally in the context of algebraic reductions of integrable equations [24], motivated by the problem of algebraic reduction of Lax pairs [28]. While the classification problem is a stand-alone one, its solution will have an impact also in applications to the theory of integrable systems and beyond. In particular, the Chevalley normal form (see Sect. 5) can be used as starting point to analyse Lax pairs and consequently associated integrable equations.

A first step towards the classification of ALiAs was presented in [24], where automorphic algebras associated with finite groups were considered. These groups are those of Klein’s classification, namely the cyclic groups $\mathbb {Z}/{N}$, the dihedral groups $\mathbb {D}_N$, the tetrahedral group $\mathbb {T}$, the octahedral group $\mathbb {O}$ and the icosahedral group $\mathbb {Y}$. In [24], the authors study automorphic algebras associated with the dihedral group $\mathbb {D}_N$, starting from the finite-dimensional algebra ${{\mathfrak {sl}}}_2(\mathbb {C})$; examples of ALiAs based on ${{\mathfrak {sl}}}_{3}(\mathbb {C}){}$ were also discussed. In [17], the authors present a classification of automorphic algebras associated with the dihedral group $\mathbb {D}_N$, where the action is inner and no summands are trivial. A further, crucial, step towards the full classification appears in [25], where the problem is formulated in a uniform way using the theory of invariants. This allows for a complete classification of ${{\mathfrak {sl}}}_2(\mathbb {C})$-based ALiAs with finite group symmetry. The new approach inspires the present results; however, the simplifying assumption that the representations of *G* acting on the spectral parameter $\lambda$ as well as on the natural representation *V* of the base Lie algebra are the same, as in [25], can no longer be made when considering higher-dimensional Lie algebras.

The aim of this paper is to present the complete classification of Automorphic Lie Algebras for the case ${{\mathfrak {g}}}={{\mathfrak {sl}}}_{n}(\mathbb {C})$ with poles at an exceptional *G*-orbit, and an inner action on ${{\mathfrak {sl}}}_n(\mathbb {C})$ that has no trivial summands. Exceptional orbits $\Gamma$ are those with less than |*G*| elements; they are labelled by ${{\mathfrak {z}}}={{\mathfrak {a}}}, {{\mathfrak {b}}}, {{\mathfrak {c}}}$, where ${{\mathfrak {a}}}, {{\mathfrak {b}}}, {{\mathfrak {c}}}$ refer to the forms with zeros at $\Gamma _{{\mathfrak {z}}}$. A key feature of this approach is the study of these algebras in the context of *classical invariant theory*. In brief, the Riemann sphere is identified with the complex projective line $\mathbb {CP}^1$ consisting of quotients ${X}\big /{Y}$ of two complex variables by setting $\lambda ={X}\big /{Y}$. Möbius transformations on $\lambda$ then correspond to linear transformations on the vector (*X*, *Y*) by the same matrix. Classical invariant theory is then used to find the *G*-invariant subspaces of $\mathbb {C}[X,Y]$-modules, where $\mathbb {C}[X,Y]$ is the ring of polynomials in *X* and *Y*. These ring modules of invariants are then localised by a choice of multiplicative set of invariants. This choice corresponds to selecting a *G*-orbit $\Gamma _{{\mathfrak {z}}}$ of poles. The set of elements in the localisation of degree zero, i.e. the set of elements which can be expressed as functions of $\lambda$, generates the ALiA. Once the algebra is computed, it is transformed into a Chevalley normal form in the spirit of the standard Chevalley basis [10]; we believe this is the most convenient form for analysis. The *isomorphism question* can finally be answered in the ${{\mathfrak {sl}}}_{n}(\mathbb {C})$ case, and a more refined isomorphism conjecture can be formulated:

*Let* *G* *and* $G'$ *be two of the groups from* $\mathbb {T}, \mathbb {O}, \mathbb {Y}$ *or* $\mathbb {D}_N$ *and let* $\Gamma _{{\mathfrak {z}}}$ *and* $\Gamma '_{{{\mathfrak {z}}}'}$ *be exceptional* *G*- *and* $G'$ *-orbits, respectively. Let* *G* *act on* ${{\mathfrak {g}}}$ *by inner automorphisms, such that* ${{\mathfrak {g}}}^G=\{0\}$, *and similarly for* $G'$ *and* ${{\mathfrak {g}}}'$. *Then, the Automorphic Lie Algebras* $({{\mathfrak {g}}}\otimes {\mathcal {M}}(\overline{\mathbb {C}}))^G_{\Gamma _{{{\mathfrak {z}}}}}$ *and* $({{\mathfrak {g}}}'\otimes {\mathcal {M}}(\overline{\mathbb {C}}))^{G'}_{\Gamma '_{{{\mathfrak {z}}}'}}$ *are isomorphic as Lie algebras if and only if* ${{\mathfrak {g}}}\cong {{\mathfrak {g}}}'$ *and* $\kappa _{{\mathfrak {z}}}=\kappa _{{{\mathfrak {z}}}'}$ (cf. Table 21—see Theorem 5.1 for the precise statement).

Classical invariant theory provides a powerful tool of analysis from the point of view of computations. Indeed, one of the obstacles to a complete classification so far was of a computational nature. There were difficulties arising on the one hand from choosing two different group representations, which implies a *ground form* of higher degree, rather than of degree two as in [25]; on the other hand, there was the intrinsic difficulty arising from the higher dimensionality of the problem (moving from ${{\mathfrak {sl}}}_2(\mathbb {C})$ to ${{\mathfrak {sl}}}_{n}(\mathbb {C})$, $n>2$).

The main results of the classification, under the conditions specified in Sect. 2.1, can be summarised as follows:

1. The long-standing isomorphism conjecture, due to Mikhailov, is now a theorem for ${{\mathfrak {g}}}={{\mathfrak {sl}}}_{n}(\mathbb {C})$ (see Theorem 5.1). The proof relies on the explicit Chevalley normal form of the algebras.
2. The number of automorphic functions present in each normal form is an invariant (see Sects. 5 and 6).

The results also suggest a natural interpretation of these algebras as finitely generated over the ring , where ${\mathbb {k}}$ is an extension of $\mathbb {Q}$ with a root of unity depending on the irreducible representations of the group *G*, and  is a *G*-automorphic function with poles at the orbit $\Gamma$ (note that the field and the automorphic function are group dependent, but we do not want to overload the notation by calling it ${\mathbb {k}}_G$; this also underlines the fact that the group dependency does not play a big role).

The alternative is to consider it as an infinite-dimensional Lie algebra over ${\mathbb {k}}$, graded by powers of , as has been done in earlier publications, cf. [25], where both approaches are used in parallel, or in [3, 24], and, in the context of KN type algebras, in [30]. While the former approach adds some computational complications, one is rewarded with classical looking Chevalley normal form results (see Sect. 5) and the Cartan matrix is the same as the one from the original Lie algebra. It is worth pointing out that in both approaches one can ask whether the ALiA can be brought into normal form, as, for instance, in the case of the Chevalley basis for simple Lie algebras over $\mathbb {C}$. As with any normal form question, one has to determine the transformation group. In the context of infinite-dimensional Lie algebras, there are now two approaches in use: (i) the graded approach, where one allows invertible linear transformations on the algebra respecting the grading. This approach in particular keeps the grading depth invariant [24]. (ii) The filtered approach, used in this paper and introduced in [25], where one allows invertible linear transformations of filtering degree 0, where the filtering is induced by the grading in the usual manner. Here the quasigrading is respected, but the grading depth may increase. Since the second group of transformations contains the first, the normal form space will be smaller. Explicitly, if the algebra $({{\mathfrak {g}}}\otimes {\mathcal {M}}(\overline{\mathbb {C}}))^G_\Gamma$ is generated by *m* matrices over the ring , then the first approach uses the transformation group $\{T\in \text {Mat}_{m\times m}(\mathbb {k})\,|\,\det (T)\in \mathbb {k}^*\}=\mathrm {GL}(\mathbb {k}^{m})$ and the second uses , namely the general linear group of the vector space $({{\mathfrak {g}}}\otimes {\mathcal {M}}(\overline{\mathbb {C}}))^G_\Gamma$.

We remark that the finite group theory used here is completely classical, with the exception of the results in Sect. 6, whereas the Lie algebra theory over a polynomial ring is slightly more modern, but it is the combination of the two that poses the central question in this paper.

Finally, it is worth pointing out that the classification is driven by computational inputs: many of the necessary computations were done using the FORM package [21], calling on GAP [8] and Singular [9].

The paper is organised as follows: in the next section, the computational challenges are presented and addressed in two ways (the difficulties arising from the increasing dimensionality of the problem are discussed in Sect. 2 but ultimately addressed in Sect. 4): first, by using classical invariant theory, thus working with polynomials in *X* and *Y* (Sect. 2.1), rather than rational functions of $\lambda$, until the very last stage when the Riemann sphere is identified with the complex projective line $\mathbb {CP}^1$ by setting $\lambda ={X}\big /{Y}$. Section 2.2 recalls the necessary background from representation theory of finite groups, considering in particular the $\mathbb {T}\mathbb {O}\mathbb {Y}$ groups. Sections 2.2 and 2.3 recall basic notions from invariant theory, such as decompositions into irreducible representations and Molien series. In Sect. 3, invariant matrices are computed by means of transvection (Sect. 3.2). The second major computational challenge of the problem is addressed in Sect. 4 introducing the concept of *matrices of invariants*, which in turn allows one to define *Chevalley normal form* for ALiAs. Normal forms for ${{\mathfrak {sl}}}_{n}(\mathbb {C})$-based ALiAs are given in Sect. 5, and Sect. 6 introduces the concept of *invariant of Automorphic Lie Algebras*. The predicting power of invariants is discussed in Conclusions (Sect. 7) where the main findings are commented upon.

## Computing Automorphic Lie Algebras

One of the obstacles to a complete classification of Automorphic Lie Algebras so far has been of computational nature: difficulties arising on the one hand from the choice of two different group representations, which implies a *ground form* of higher degree, rather than of degree two as in [25]. On the other hand, the intrinsic difficulty arising from the higher dimensionality of the problem, moving from ${{\mathfrak {sl}}}_2(\mathbb {C})$ to ${{\mathfrak {sl}}}_{n}(\mathbb {C})$, $n>2$. These difficulties are overcome here in two ways: first, by using classical invariant theory, thus working with polynomials in *X* and *Y* rather than rational functions of $\lambda$, until the very last stage when the Riemann sphere is identified with the complex projective line $\mathbb {CP}^1$ by setting $\lambda ={X}\big /{Y}$. This allows us a better control of the degrees of the invariants at each step of the computation, and it enables the use of Molien’s theory to predict the degree of the invariants and to check the outcome of the computations as well. Working over $\mathbb {C}[X,Y]$ allows us also to use transvectants, an easy to implement computational tool in classical invariant theory (see Sect. 3.2). The difficulty arising from the higher dimensionality of the problem is instead dealt with introducing *matrices of invariants* (see Sect. 4), which are computationally very effective. They are defined by considering the action of invariant matrices on invariant vectors, by multiplication. The description of the invariant matrices in terms of this action yields greatly simplified matrices, whose entries are indeed *G*-invariant. The map to matrices of invariants preserves the structure constants of the Lie algebra. We emphasise that the matrices of invariants are not invariant under the usual group action, because they are expressed in a $\lambda$-dependent basis that trivialises the conjugation action on the matrices, leaving only the action on the spectral parameter $\lambda$ (see next section).

We start by defining *Polynomial Automorphic Lie Algebras*.

### Polynomial Automorphic Lie Algebras

Let *G* be a finite group and let $\sigma$ be a faithful, projective *G*-representation:

$$\begin{aligned} \sigma :G\rightarrow \text {GL}_2(\mathbb {C}). \end{aligned}$$

This restricts *G* to the groups

$$\begin{aligned} \mathbb {Z}/N,\;\mathbb {D}_N,\;\mathbb {T},\;\mathbb {O},\;\mathbb {Y}\end{aligned}$$

of Klein’s classification [13, 14] where $\mathbb {Z}/N$ is the cyclic group, $\mathbb {D}_N$ the dihedral group, $\mathbb {T}$ the tetrahedral group, $\mathbb {O}$ the octahedral group and $\mathbb {Y}$ the icosahedral group. In this paper, we focus on the exceptional cases (since they are not part of infinite families), the $\mathbb {T}\mathbb {O}\mathbb {Y}$ groups. The $\mathbb {D}_N$-classification has been presented in [17], both for generic and exceptional *G*-orbits, since the $\mathbb {D}_N$ computations can be done explicitly without the use of a computer. In addition, this is the only nonabelian group in Klein’s classification whose order depends on *N*, which is a complication from a computational point of view, and we prefer to keep it separate.

Let *V* be a finite-dimensional vector space, let $\tau :G\rightarrow \text {PGL}(V)$ be an irreducible *G*-representation. Consider the Lie algebra

$$\begin{aligned} {{\mathfrak {g}}}(V)\otimes \mathbb {C}[X,Y]\, \end{aligned}$$

where ${{\mathfrak {g}}}(V)$ is a complex Lie algebra in ${{\mathfrak {gl}}}(V)$ and $\mathbb {C}[X,Y]$ is the ring of polynomials in *X* and *Y*. The representations $\sigma$ and $\tau$ induce a *G*-action on ${{\mathfrak {g}}}(V)\otimes \mathbb {C}[X,Y]$ (see [34, Section 1.5, 1.6]) by identifying ${{\mathfrak {gl}}}(V)=V\otimes V^{*}$, where $V^{*}$ is the dual space,

$$\begin{aligned} g\cdot \big (M\otimes p(X,Y)\big )=\tau (g) M \tau (g^{-1})\otimes p\big (\sigma (g^{-1})(X,Y)\big ). \end{aligned}$$

Notice that this defines a Lie algebra automorphism of ${{\mathfrak {g}}}(V)\otimes \mathbb {C}[X,Y]$. This is the general set-up for the inner reduction group where the base Lie algebra has no trivial summands in the following sense. For every homomorphism $\rho :G\rightarrow \mathrm {Int}({{\mathfrak {g}}}(V))$, there exists a homomorphism $\tau :G\rightarrow \mathrm {PGL}(V)$ such that $\rho =\mathrm {Ad}\circ \tau$. Moreover, ${{\mathfrak {g}}}(V)^{\rho (G)}=\{0\}$ if and only if $\tau$ is irreducible.

#### *Remark 2.1*

Notice there are monomorhpisms $G\in {\text {Aut}}({{\mathfrak {g}}}\otimes {\mathcal {M}}(\overline{\mathbb {C}}))$ not covered by this description. Indeed, if $n=2$, then $\mathrm {Aut}\left( {{\mathfrak {sl}}}_n(\mathbb {C}) \right) =\mathrm {Int}\left( {{\mathfrak {sl}}}_n(\mathbb {C}) \right)$. If $n>2$, then $\mathrm {Aut}\left( {{\mathfrak {sl}}}_n(\mathbb {C}) \right) / \mathrm {Int}\left( {{\mathfrak {sl}}}_n(\mathbb {C}) \right) \cong \mathbb {Z}/{2}$. Therefore, if $\rho (G)\rightarrow \mathrm {Aut}\left( {{\mathfrak {sl}}}_n(\mathbb {C}) \right)$, then $\rho (G)\cap \mathrm {Int}\left( {{\mathfrak {sl}}}_n(\mathbb {C}) \right)$ is a normal subgroup of $\rho (G)$ of index 1 or 2. For cases of $\rho (G)$ that do not have an index 2 normal subgroup, the action will be inner and the above set-up is complete. These groups include the tetrahedral and icosahedral groups, as well as cyclic groups of odd order. Polyhedral groups that do have a normal subgroup of index 2 are cyclic groups of even order, dihedral groups and the octahedral group. One can show that all these groups have actions on ${{\mathfrak {sl}}}_n(\mathbb {C})$, which include outer automorphisms. Examples of the dihedral case are studied in [24].

The analysis of all admissible automorphisms in $\text {Aut}({{\mathfrak {g}}}\otimes {\mathcal {M}}(\overline{\mathbb {C}}))$ given a Lie algebra ${{\mathfrak {g}}}$ is a very interesting one, and it is left for further investigation.

#### **Definition 2.1**

Let *V* be a *G*-module. An element $v\in V$ is called $\chi$ **-relative invariant** if there exists a homomorphism $\chi \,:\, G\rightarrow \mathbb {C}^{*}$, the multiplicative group of $\mathbb {C}$, such that $g\,v=\chi (g)\, v$. If $\chi$ is trivial, then *v* is called **invariant**. The space of $\chi$-relative invariants in *V* will be denoted by $V^{\chi }_G$ (or simply $V^{\chi }$ if there is no confusion with respect to the group), the space generated by all relative invariants by $V_G$ and the subspace of invariants by $V^G$.

#### *Remark 2.2*

An example of a homomorphism $\chi \,:\, G\rightarrow \mathbb {C}^{*}$ is the determinant of a *G*-representation $\rho$, $\Delta _\rho (g)=\det \rho (g)$.

#### **Definition 2.2**

The algebra $({{\mathfrak {g}}}(V)\otimes \mathbb {C}[X,Y])^G$ defines a **Polynomial Automorphic Lie Algebra** based on ${{\mathfrak {g}}}(V)$ cf. [25].

Our first goal will be to compute Polynomial ALiAs, $({{\mathfrak {g}}}(V)\otimes \mathbb {C}[X,Y])^G$, where *G* is one of the $\mathbb {T}\mathbb {O}\mathbb {Y}$ groups.

In the following, we fix a group *G* and a natural representation $\sigma$ and vary $\tau$ through all possible irreducible projective *G*-representations.

### Irreducible Representations

We recall that our ultimate goal is to construct and classify all Automorphic Lie Algebras, $({{\mathfrak {g}}}(V)\otimes {\mathcal {M}}(\overline{\mathbb {C}}))^G_\Gamma$, where *G* is a finite group, ${\mathcal {M}}(\overline{\mathbb {C}})$ is the field of meromorphic functions on the Riemann sphere and where $\Gamma \subset \overline{\mathbb {C}}$ is a *G*-orbit. Using the identification $\lambda ={X}\big /{Y}\in \mathbb {CP}^1$, the space ${\mathcal {M}}(\overline{\mathbb {C}})$ is identified with the space of quotients of two homogeneous polynomials in *X* and *Y* of the same degree. Möbius transformations on $\lambda$ correspond to linear transformations on *X* and *Y* by the same matrix. Moreover, two matrices yield the same Möbius transformation if and only if they are scalar multiples of one another. Therefore, in order to cover all possibilities, we allow the action on *X* and *Y* to be projective. We recall that a faithful projective representation $\sigma$ of *G* in $\mathbb {C}^2$ is a mapping from *G* to $\mathrm {GL}_2(\mathbb {C})$ obeying the following

1 $$\begin{aligned} \sigma (g)\,\sigma (h)=c(g,h)\,\sigma (gh),\quad \forall g,h\in G, \end{aligned}$$

where $c(g,h)\,:\; G\times G\rightarrow \mathbb {C}^{*}$ in (1) is a 2-cocycle over $\mathbb {C}^{*}$ (see for example [39]), satisfying the cocycle identity

$$\begin{aligned} c(x,y)c(xy,z)=c(y,z)c(x,yz). \end{aligned}$$

If the cocycle is trivial, the projective representation $\sigma$ is a representation. Projective representations of *G* correspond to representations of any Schur cover of *G*. We define the Schur cover $G^\flat$ of *G* in $\mathrm {SL}_2(\mathbb {C})$ as the preimages of $G\subset \mathrm {PSL}_2(\mathbb {C})$, under the canonical projection $\pi :\mathrm {SL}_2(\mathbb {C})\rightarrow \mathrm {PSL}_2(\mathbb {C})$:

$$\begin{aligned} G^\flat =\pi ^{-1}G. \end{aligned}$$

Alternatively, this group can be defined by the presentation

$$\begin{aligned} G^\flat =\left\langle g_{{\mathfrak {a}}}, g_{{\mathfrak {b}}}, g_{{\mathfrak {c}}}\;|\;g_{{\mathfrak {a}}}^{d_G}=g_{{\mathfrak {b}}}^3=g_{{\mathfrak {c}}}^2=g_{{\mathfrak {a}}}g_{{\mathfrak {b}}}g_{{\mathfrak {c}}}\right\rangle , \end{aligned}$$

cf. [38], where $d_G=3,4$ and 5 for $\mathbb {T}$, $\mathbb {O}$ and $\mathbb {Y}$, respectively. We can readily see that $g_{{\mathfrak {a}}}g_{{\mathfrak {b}}}g_{{\mathfrak {c}}}$ is a central element because it commutes with each generator, e.g. $g_{{\mathfrak {a}}}(g_{{\mathfrak {a}}}g_{{\mathfrak {b}}}g_{{\mathfrak {c}}})=g_{{\mathfrak {a}}}g_{{\mathfrak {a}}}^{d_G}=g_{{\mathfrak {a}}}^{d_G} g_{{\mathfrak {a}}}=(g_{{\mathfrak {a}}}g_{{\mathfrak {b}}}g_{{\mathfrak {c}}})g_{{\mathfrak {a}}}$. If $G^\flat$ is nonabelian, then this is the only nontrivial central element and represented by minus the identity matrix in $\mathrm {SL}_2(\mathbb {C})$. In particular, it has order 2 and the projection $\pi$ maps it to the identity. Another presentation is given by

$$\begin{aligned} r=g_{{\mathfrak {a}}},\qquad s=g_{{\mathfrak {c}}}. \end{aligned}$$

Then $g_{{\mathfrak {b}}}=g_{{\mathfrak {a}}}^{-1}(g_{{\mathfrak {a}}}g_{{\mathfrak {b}}}g_{{\mathfrak {c}}})g_{{\mathfrak {c}}}^{-1}=g_{{\mathfrak {a}}}^{-1}(g_{{\mathfrak {c}}}^2)g_{{\mathfrak {c}}}^{-1}=g_{{\mathfrak {a}}}^{-1}g_{{\mathfrak {c}}}=r^{-1}s$ and we obtain

$$\begin{aligned} G^\flat =\left\langle r,s\;|\;r^{d_G}=\left( r^{-1}s\right) ^3=s^2\right\rangle . \end{aligned}$$

In Appendix 1, we give an explicit construction of the Schur cover $G^\flat$ we work with, for completeness.

From a computational point of view, it is more convenient to work with representations, rather than projective representations. For example, in order to use GAP to compute generating elements, character tables (Sects. 2.2.2–2.2.4) and Molien functions (Sect. 2.3), one needs to replace the projective representation by a representation.

Linear representations of $\mathbb {T}^\flat$, $\mathbb {O}^\flat$, $\mathbb {Y}^\flat$ can be easily computed by GAP (see Sects. 2.2.2–2.2.4 for further details); in what follows we label irreducible representations (irreps) by $G^\flat _i$, where *G* is one of the $\mathbb {T}\mathbb {O}\mathbb {Y}$ groups, and we drop $\flat$ when the representation is also a linear representation of *G*. We denote this set as $\text {Irr}(G^\flat )$. The representations with a $\flat$-index are those with nontrivial cocycle (see Tables 1, 2, 3); these are the representations, which are not linear representations of *G*.

#### **Definition 2.3**

(*Natural representation*) A monomorphism

$$\begin{aligned} \sigma :G^\flat \rightarrow \text {SL}_2(\mathbb {C}) \end{aligned}$$

is called a **natural representation**.

The chosen natural representations of the $\mathbb {T}\mathbb {O}\mathbb {Y}$ groups are underlined in Tables 1, 2 and 3.

#### Dynkin Diagrams of the Irreducible Representations

Before proceeding with a list of irreducible $G^\flat$-representations, let us recall here some results from [36]. Let $\mathbb {T}^\flat ,\mathbb {O}^\flat ,\mathbb {Y}^\flat$ be the double covers of the $\mathbb {T}\mathbb {O}\mathbb {Y}$ groups; they are characterised by the solutions of the equation

2 $$\begin{aligned} \frac{1}{a}+\frac{1}{b}+\frac{1}{c}=1\,\quad a,b,c\in \mathbb {N}. \end{aligned}$$

The solutions are well known, and they are (6, 3, 2) for $\mathbb {Y}^\flat$, (4, 4, 2) for $\mathbb {O}^\flat$ and (3, 3, 3) for $\mathbb {T}^\flat$, up to permutation.

We will closely follow the notations in [36], so for the purpose of the diagrams we rename the natural representation $\sigma$ with *x* and denote by $x_h$ the *h*-th symmetric power of *x*. Notice that $x_0$ is the trivial representation and $x_1=x$ the natural representation. The Clebsch–Gordan formula from classical invariant theory is

3 $$\begin{aligned} x\otimes x_h =x_{h-1}\oplus x_{h+1}\,\quad h\ge 1. \end{aligned}$$

**Fig. 1:**
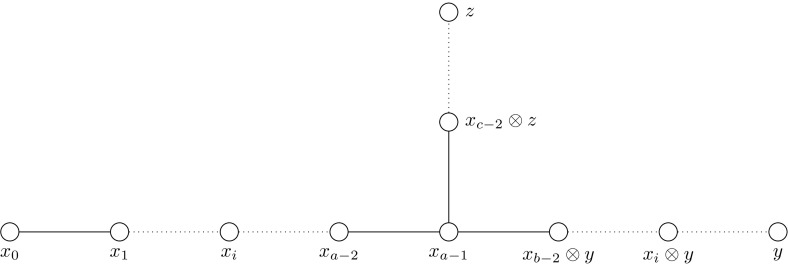
Affine Dynkin diagrams of $G^\flat$, where *G* is one of the $\mathbb {T}\mathbb {O}\mathbb {Y}$ groups. The dimensions of the irreducibles are $1, 2,\ldots , a$; ${a}\big /{b},2{a}\big /{b},\ldots ,(b-1){a}\big /{b}$; ${a}\big /{c},2{a}\big /{c},\ldots ,(c-1){a}\big /{c}$

Let $x_0$, *y* and *z* be the three different endpoints of the Dynkin diagram of affine type (this is also called extended Dynkin diagram, as it contains the trivial representation $x_0$—see Fig. 1). The diagram is formed by taking the irreducible representations as nodes. Every representation is connected to those irreducible representations that occur in the decomposition of its tensor product with the natural representation into irreducible representations. Let $a\ge 2$ be such that $x_0$, $x_1$,...,$x_{a-1}$ are irreducible as $G^\flat$-modules and $x_a$ is not, then $x_{a-1}$ is called branch point (of the Dynkin diagram). There are integers $b, c\ge 2$ such that the two other branches of the Dynkin diagram are given by $y, x_1 y,\ldots ,x_{b-2} y$ and $z,x_1 z,\ldots ,x_{c-2}z$, respectively, and it follows that $x_{a}$ splits into two irreducibles according to the rule

$$\begin{aligned} x\otimes x_{a-1}=x_{a-2}\oplus x_{a}=x_{a-2}\oplus x_{b-2}\otimes y\oplus x_{c-2}\otimes z\, \end{aligned}$$

(see [36] for details). The branch point is characterised by $x_{a-1}=x_{b-1}\otimes y=x_{c-1}\otimes z$ and (*a*, *b*, *c*) satisfy Eq. (2).

#### Tetrahedral Group $\mathbb {T}$

A regular tetrahedron is a Platonic solid composed of four equilateral triangular faces, three of which meet at each vertex. It has four vertices and six edges. A regular tetrahedron has twelve rotational (or orientation-preserving) symmetries; the set of orientation-preserving symmetries forms a group referred to as $\mathbb {T}$, isomorphic to the alternating subgroup ${\mathcal {A}}_4$. As an abstract group, it is generated by two elements, $r$ and $s$, satisfying the identities $r^{3}=s^{2}=(r\,s)^3=id.$

In Table 1, the character table of the Schur cover $\mathbb {T}^\flat =\langle r,s\;|\;r^{3}=(r^{-1}s)^3=s^2\rangle$ in SL$_2 (\mathbb {C})$ (see Sect. 2.2) is given. The first column contains the seven irreducible representations of $\mathbb {T}^\flat$; they can be obtained by e.g. GAP [8]; the irreducible representation $\mathbb {T}_4^\flat$ is the *natural representation* (see Definition 2.3). The representations with a $\flat$-index are those with nontrivial cohomology (see Appendix 1); the $\flat$ is dropped when the representation is also a linear representation of $\mathbb {T}$. The second column contains the same representations in the language of [36] to allow drawing the Dynkin diagram as in Sect. 2.2.1. The next columns list the conjugacy classes and the corresponding values of the characters, following the GAP notation, where $A = \omega _3^2$, $/A=\omega _3$. Notice that the trace of *id* (the only element in [*id*]) is the dimension of the representation. Here, and in what follows, $\omega _n=\exp {{2\pi i}\big /{n}}$, so $\omega _3$ is a primitive cubic root of unity. The penultimate column contains determinants of the representation (see Remark 2.2). Determinants have been included since they suggest the pairing of relative invariants in order to get invariants from transvection (Sect. 3.2) and (for future reference) play a role in the determination of the building blocks of ${{\mathfrak {sl}}}(V)$. Finally, the last column contains the value of the Frobenius–Schur indicator $\iota$, computed by $\iota _\chi =\frac{1}{|G|}\sum _{g\in G}\chi (g^2)$. Complex irreducible representations with Frobenius–Schur indicator 1, 0 or $-1$ are, respectively, known as representations of real type, complex type or quaternionic type [7]. This last column is included here purely for future reference, as it gives information about the actions on ${{\mathfrak {so}}}$ and ${{\mathfrak {sp}}}$.

**Table 1:** Character table for $\mathbb {T}^\flat$, $A = \omega _3^2$, $/A=\omega _3$, in GAP notation

irrep	Dynkin	[*id*]	$$ [(r^{-1}s)^2]$$	[*s*]	$$ [s^2]$$	$$[r^2]$$	[*r*]	$$ [r^{-1}s]$$	$$\Delta $$	$$\iota $$
$\mathbb {T}_1$	$x_0$	1	1	1	1	1	1	1	$\mathbb {T}_1$	1
$\mathbb {T}_2$	*y*	1	*A*	1	1	/*A*	*A*	/*A*	$\mathbb {T}_2$	0
$\mathbb {T}_3$	*z*	1	/*A*	1	1	*A*	/*A*	*A*	$\mathbb {T}_3$	0
$\underline{\mathbb {T}_4^\flat }$	$x_1$	2	$-$1	0	$-$2	$-$1	1	1	$\mathbb {T}_1$	$-1$
$\mathbb {T}_5^\flat$	$x_1\otimes z$	2	$-$/*A*	0	$-$2	$-$ *A*	/*A*	*A*	$\mathbb {T}_2$	0
$\mathbb {T}_6^\flat$	$x_1\otimes y$	2	$-$ *A*	0	$-$2	$-$/*A*	*A*	/*A*	$\mathbb {T}_3$	0
$\mathbb {T}_7$	$x_2$	3	0	$-1$	3	0	0	0	$\mathbb {T}_1$	1

A concrete projective representation of $\mathbb {T}_4^\flat$ is given by

4 $$\begin{aligned} \sigma \left( r^2\right) = \begin{pmatrix} \omega _3^2&{}\quad 0 \\ 0 &{}\quad \omega _3 \end{pmatrix}, \quad \sigma (s)=\frac{1}{3}(1+2\omega _3) \begin{pmatrix} -1&{}\quad -1\\ -2 &{}\quad 1 \end{pmatrix}. \end{aligned}$$

Note that the matrix group is independent of the choice of generators; hence, this choice is irrelevant. We present here the generators used in the computations. Table 1 suggests the following field extension: ${\mathbb {k}}=\mathbb {Q}[\omega _3]/(1+\omega _3+\omega _3^2)$; the nonzero elements are denoted by ${\mathbb {k}}^*$.

#### Octahedral Group $\mathbb {O}$

A regular octahedron is a Platonic solid composed of eight equilateral triangles, four of which meet at each vertex; it has six vertices and eight edges. A regular octahedron has twenty-four rotational (or orientation-preserving) symmetries. A cube has the same set of symmetries, since it is its dual. The group of orientation-preserving symmetries is denoted by $\mathbb {O}$, and it is isomorphic to ${\mathcal {S}}_4$, or the group of permutations of four objects, since there is exactly one such symmetry for each permutation of the four pairs of opposite sides of the octahedron. As an abstract group, it is generated by two elements, $r$ and $s$, satisfying the identities $r^{4}=s^{2}=(r\,s)^3=id.$

**Table 2:** Character table for $\mathbb {O}^\flat$, $A = -\omega _8+\omega _8^3=-\sqrt{2}$, in GAP notation

irrep	Dynkin	[*id*]	[*s*]	$[(r^{-1}s)^2]$	$[r^2]$	$[s^2]$	[*r*]	[*rs*]	$[r^3]$	$\Delta$	$\iota$
$\mathbb {O}_1$	$x_0$	1	1	1	1	1	1	1	1	$\mathbb {O}_1$	1
$\mathbb {O}_2$	*y*	1	$-$1	1	1	1	$-$1	1	$-$1	$\mathbb {O}_2$	1
$\mathbb {O}_3$	*z*	2	0	$-$1	2	2	0	$-$1	0	$\mathbb {O}_2$	1
$\underline{\mathbb {O}}_4^\flat$	$x_1$	2	0	$-$1	0	$-$2	*A*	1	$-$ *A*	$\mathbb {O}_1$	$-1$
$\mathbb {O}_5^\flat$	$x_1\otimes y$	2	0	$-$1	0	$-$2	$-$ *A*	1	*A*	$\mathbb {O}_1$	$-1$
$\mathbb {O}_6$	$x_2\otimes y$	3	1	0	$-$1	3	$-$1	0	$-$1	$\mathbb {O}_2$	1
$\mathbb {O}_7$	$x_2$	3	$-$1	0	$-$1	3	1	0	1	$\mathbb {O}_1$	1
$\mathbb {O}_8^\flat$	$x_3$	4	0	1	0	$-$4	0	$-$1	0	$\mathbb {O}_1$	$-1$

The character table of the Schur cover $\mathbb {O}^\flat =\langle r,s\;|\;r^{4}=(r^{-1}s)^3=s^2\rangle$ in SL$_2 (\mathbb {C})$ (see Sect. 2.2) is given in Table 2. The irreducible representation $\mathbb {O}_4^\flat$ is the natural representation that we will use.

The concrete projective representation we work with is given by

5 $$\begin{aligned} \sigma \left( (r^{-1}s)^2\right)= & {} \begin{pmatrix} -\omega _{24}^4 &{}\quad 0\\ 0&{}\quad -1+\omega _{24}^4 \end{pmatrix},\nonumber \\ \sigma (r)= & {} \frac{1}{3}\begin{pmatrix} -2\omega _{24} -\omega _{24}^3+\omega _{24}^5-\omega _{24}^7&{}\quad \omega _{24}^2+\omega _{24}^6\\ -\omega _{24}^2+2\omega _{24}^6&{}\quad -\omega _{24}-2\omega _{24}^3+2\omega _{24}^5+\omega _{24}^7 \end{pmatrix}\nonumber \\ \end{aligned}$$

As in the previous case, the chosen field is determined by the occurrence of roots of unity in the representation matrices. In the $\mathbb {O}^\flat$ case, $\omega _{24}$ occurs. The minimal polynomial is then the one for $\omega _6$ but expressed for $\omega _{24}$. Hence, the field extension in this case is ${\mathbb {k}}=\mathbb {Q}[\omega _{24}]/(\omega ^8_{24}-\omega ^4_{24}+1)$.

#### Icosahedral Group $\mathbb {Y}$

An icosahedron is a convex regular polyhedron (a Platonic solid) with twenty triangular faces, thirty edges and twelve vertices. A regular icosahedron has sixty rotational (or orientation-preserving) symmetries; the set of orientation-preserving symmetries forms a group referred to as $\mathbb {Y}$; $\mathbb {Y}$ is isomorphic to ${\mathcal {A}}_5$, the alternating group of even permutations of five objects. As an abstract group, it is generated by two elements, $r$ and $s$, satisfying the identities $r^{5}=s^{2}=(r\,s)^3=id.$

The Schur cover $\mathbb {Y}^\flat =\langle r,s\;|\;r^{5}=(r^{-1}s)^3=s^2\rangle$ in $SL_2 (\mathbb {C})$ (see Sect. 2.2) has the following character Table 3.

The concrete projective representation we work with is given by

6 $$\begin{aligned} \sigma (r^4)= \begin{pmatrix}\omega _5^4 &{}\quad 0 \\ 0 &{} \omega _5 \end{pmatrix}, \quad \sigma (s)=\frac{1}{5} \begin{pmatrix} -2-4\omega _5-\omega _5^2 -3\omega _5^3 &{} 3+\omega _5-\omega _5^2-3\omega _5^3 \\ -3-\omega _5+\omega _5^2-2\omega _5^3 &{} 2+4 \omega _5+\omega _5^2+3\omega _5^3 \end{pmatrix}\nonumber \\ \end{aligned}$$

and $${\mathbb {k}}=\mathbb {Q}[\omega _5]/(1+\omega _5+\omega _5^2+\omega _5^3+\omega _5^4)$$.

#### Decomposition of ${{\mathfrak {sl}}}(V)$ into Irreducible Representations

We compute the decomposition of ${{\mathfrak {sl}}}(V_j)\cong V_j\otimes V_j^*-V_1$ into irreducible representations using GAP, where $V_1$ is the trivial representation and list them in Tables 4, 5 and 6. This is the first moment we specialise to ${{\mathfrak {sl}}}(V)$; we remark that similar decompositions exist for ${{\mathfrak {so}}}(V)$ and ${{\mathfrak {sp}}}(V)$, and this paper contains all the necessary information to analyse these cases as well. The irreducible representations $V_j$ are labelled using the group name, so $\mathbb {T}_1$ corresponds to the first irreducible representation in the list of $\mathbb {T}^\flat$ (see Tables 1, 2, 3).

**Table 3:** Character table for $\mathbb {Y}^\flat$, $A = \omega _5+\omega _5^4$, $*A=1-A=A^2=-1/A$, in GAP notation

irrep	Dynkin	[*id*]	$[r^4]$	$[r^2]$	$[r^{-1}s]$	[*s*]	$[(r^{-1}s)^2]$	[*r*]	$[s^2]$	$[r^3]$	$\Delta$	$\iota$
$\mathbb {Y}_1$	$x_0$	1	1	1	1	1	1	1	1	1	$\mathbb {Y}_1$	1
$\underline{\mathbb {Y}}_2^\flat$	$x_1$	2	*A*	*A	1	0	$-$1	$-$ *A*	$-$2	$-$*A	$\mathbb {Y}_1$	$-1$
$\mathbb {Y}_3^\flat$	*y*	2	*A	*A*	1	0	$-$1	$-$*A	$-$2	$-$ *A*	$\mathbb {Y}_1$	$-1$
$\mathbb {Y}_4$	*z*	3	$-$*A	$-$ *A*	0	$-$1	0	$-$*A	3	$-$ *A*	$\mathbb {Y}_1$	1
$\mathbb {Y}_5$	$x_2$	3	$-$ *A*	$-$*A	0	$-$1	0	$-$ *A*	3	$-$*A	$\mathbb {Y}_1$	1
$\mathbb {Y}_6$	$x_1 \otimes y$	4	$-$1	$-$1	1	0	1	$-$1	4	$-$1	$\mathbb {Y}_1$	1
$\mathbb {Y}_7^\flat$	$x_3$	4	$-$1	$-$1	$-$1	0	1	1	$-$4	1	$\mathbb {Y}_1$	$-1$
$\mathbb {Y}_8$	$x_4$	5	0	0	$-$1	1	$-$1	0	5	0	$\mathbb {Y}_1$	1
$\mathbb {Y}_9^\flat$	$x_5$	6	1	1	0	0	0	$-$1	$-$6	$-$1	$\mathbb {Y}_1$	$-1$

**Table 4:** Decomposition of ${{\mathfrak {sl}}}(\mathbb {T}_j^\flat )$

${{\mathfrak {sl}}}(\mathbb {T}_j)$	dim	Decomposition
${{\mathfrak {sl}}}\left( \mathbb {T}_4^\flat \right)$	3	$\mathbb {T}_7$
${{\mathfrak {sl}}}\left( \mathbb {T}_5^\flat \right)$	3	$\mathbb {T}_7$
${{\mathfrak {sl}}}\left( \mathbb {T}_6^\flat \right)$	3	$\mathbb {T}_7$
${{\mathfrak {sl}}}(\mathbb {T}_7)$	8	$\mathbb {T}_2\oplus \mathbb {T}_3\oplus 2\mathbb {T}_7$

**Table 5:** Decomposition of ${{\mathfrak {sl}}}(\mathbb {O}_j^\flat )$

${{\mathfrak {sl}}}(\mathbb {O}_j)$	dim	Decomposition
${{\mathfrak {sl}}}(\mathbb {O}_3)$	3	$\mathbb {O}_{2}\oplus \mathbb {O}_3$
${{\mathfrak {sl}}}\left( \mathbb {O}_4^\flat \right)$	3	$\mathbb {O}_7$
${{\mathfrak {sl}}}\left( \mathbb {O}_5^\flat \right)$	3	$\mathbb {O}_7$
${{\mathfrak {sl}}}(\mathbb {O}_6)$	8	$\mathbb {O}_3\oplus \mathbb {O}_6\oplus \mathbb {O}_7$
${{\mathfrak {sl}}}(\mathbb {O}_7)$	8	$\mathbb {O}_3\oplus \mathbb {O}_6\oplus \mathbb {O}_7$
${{\mathfrak {sl}}}\left( \mathbb {O}_8^\flat \right)$	15	$\mathbb {O}_2\oplus \mathbb {O}_3\oplus 2\mathbb {O}_6\oplus 2\mathbb {O}_7$

**Table 6:** Decomposition of ${{\mathfrak {sl}}}(\mathbb {Y}_j^\flat )$

${{\mathfrak {sl}}}(\mathbb {Y}_j)$	dim	Decomposition
${{\mathfrak {sl}}}\left( \mathbb {Y}_2^\flat \right)$	3	$\mathbb {Y}_{5}$
${{\mathfrak {sl}}}\left( \mathbb {Y}_3^\flat \right)$	3	$\mathbb {Y}_{4}$
${{\mathfrak {sl}}}(\mathbb {Y}_4)$	8	$\mathbb {Y}_4\oplus \mathbb {Y}_8$
${{\mathfrak {sl}}}(\mathbb {Y}_5)$	8	$\mathbb {Y}_5\oplus \mathbb {Y}_8$
${{\mathfrak {sl}}}(\mathbb {Y}_6)$	15	$\mathbb {Y}_4\oplus \mathbb {Y}_5\oplus \mathbb {Y}_6\oplus \mathbb {Y}_8$
${{\mathfrak {sl}}}\left( \mathbb {Y}_7^\flat \right)$	15	$\mathbb {Y}_4\oplus \mathbb {Y}_5\oplus \mathbb {Y}_6\oplus \mathbb {Y}_8$
${{\mathfrak {sl}}}(\mathbb {Y}_8)$	24	$\mathbb {Y}_4\oplus \mathbb {Y}_5\oplus 2\mathbb {Y}_6\oplus 2\mathbb {Y}_8$
${{\mathfrak {sl}}}\left( \mathbb {Y}_9^\flat \right)$	35	$2\mathbb {Y}_4\oplus 2\mathbb {Y}_5\oplus 2\mathbb {Y}_6\oplus 3\mathbb {Y}_8$

### Molien Functions

In the search for invariants in ${{\mathfrak {sl}}}(V)\otimes \mathbb {C}[X,Y]$, we use the decomposition of ${{\mathfrak {sl}}}(V)$ in the irreducible representations listed in Tables 4, 5, and 6:

$$\begin{aligned} {{\mathfrak {sl}}}(V)=\bigoplus _k \langle {{\mathfrak {sl}}}(V),V_k \rangle V_k. \end{aligned}$$

This reduces the problem to describing $(V_k \otimes \mathbb {C}[X,Y])^{G^\flat }$. The generating functions of invariants in $V_k \otimes \mathbb {C}[X,Y]$ can be computed using the following theorem (see [35, Section 4.3]).

#### **Theorem 2.1**

(Molien, [29]) Let $\sigma : \, G^{\flat } \hookrightarrow \mathrm {GL}_2(\mathbb {C})$ be a representation defining an action of $G^\flat$ on $\mathbb {C}[X,Y]$ by $g\cdot p(X,Y)=p(\sigma (g^{-1})(X,Y))$, $g\in G^{\flat }$, $p(X,Y)\in \mathbb {C}[X,Y]$, and let $\chi _k$ be the character of $V_k$. Then, the Poincaré series of invariants in $V_k \otimes \mathbb {C}[X,Y]$ is given by

7 $$\begin{aligned} M\left( ({V_k}\otimes \mathbb {C}[X,Y])^{G^{\flat }},t\right) =\frac{1}{|G^{\flat }|}\sum _{g\in G^{\flat }} \frac{\chi _k (g)}{\det (1-\sigma (g^{-1})\,t)}. \end{aligned}$$

We will call this the Molien function of the irreducible representation $V_k$.

Recall the irreducible representations $x_i$, $i=0,\ldots ,a-1$, $x_i\otimes y$, $i=0,\ldots ,b-2$ and $x_i\otimes z$, $i=0,\ldots ,c-2$ from Sect. 2.2.1. The following holds (see [36])

8 $$\begin{aligned} M(\square ,t)=\frac{N(\square ,t)}{(1-t^{2a})(1-t^{4a-4})} \end{aligned}$$

with $N(\square ,t)$ defined by

9 $$\begin{aligned} N\left( \left( {x_i}\otimes \mathbb {C}[X,Y]\right) ^{G^{\flat }},t\right)= & {} t^i +t^{6a -6 -i}+\left( t^{2a-i}+t^{4a-4-i}\right) \frac{1-t^{2i}}{1-t^2},\nonumber \\&\quad i=0,\ldots ,a-1,\nonumber \\ N\left( \left( {x_i\otimes y}\otimes \mathbb {C}[X,Y]\right) ^{G^{\flat }},t\right)= & {} t^{a+b-i-2} \left( 1+t^{2a -2}\right) \frac{1-t^{2a}}{1-t^{2b}}\frac{1-t^{2i+2}}{1-t^2},\nonumber \\&\quad i=0,\ldots ,b-2,\nonumber \\ N\left( \left( {x_i\otimes z}\otimes \mathbb {C}[X,Y]\right) ^{G^{\flat }},t\right)= & {} t^{a+c-i-2} \left( 1+t^{2a -2}\right) \frac{1-t^{2a}}{1-t^{2c}}\frac{1-t^{2i+2}}{1-t^2},\nonumber \\&\quad i=0,\ldots ,c-2. \end{aligned}$$

#### *Example 2.1*

Consider the Poincaré series of invariants in $\mathbb {T}_1\otimes \mathbb {C}[X,Y]$, with $x_0$ in the notation above. The affine Dynkin diagram of $\mathbb {T}^\flat$, where $\mathbb {T}_1$ coincides with $x_0$, is 
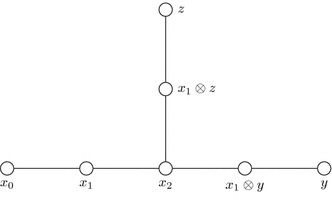
 and it is characterised by $(a=3,b=3,c=3)$ (see Sect. 2.2.1). It follows from (9) that

$$\begin{aligned} N\left( (x_0\otimes \mathbb {C}[X,Y])^{\mathbb {T}^\flat },t\right) =1+t^{12}, \end{aligned}$$

thus

$$\begin{aligned} M\left( (\mathbb {T}_1\otimes \mathbb {C}[X,Y])^{\mathbb {T}^\flat },t\right) =\frac{1+t^{12}}{(1-t^6)(1-t^8)}. \end{aligned}$$

**Table 7:** Molien functions of the irreducible representations: $M((\mathbb {T}_k\otimes \mathbb {C}[X,Y])^{\mathbb {T}^\flat },t)$

irrep	Dynkin	dim	Molien function numerator *N*
$\mathbb {T}_1$	$x_0$	1	$1+t^{12}$
$\mathbb {T}_2$	*y*	1	$t^{4}+t^{8}$
$\mathbb {T}_3$	*z*	1	$t^{4}+t^{8}$
$\underline{\mathbb {T}}_{4}^\flat$	$x_1$	2	$t+t^5+t^{7}+t^{11}$
$\mathbb {T}_{5}^\flat$	$x_1\otimes z$	2	$t^{3}+t^5+t^{7}+t^{9}$
$\mathbb {T}_{6}^\flat$	$x_1\otimes y$	2	$t^{3}+t^5+t^{7}+t^{9}$
$\mathbb {T}_{7}$	$x_2$	3	$t^2+t^4+2t^{6}+t^8+t^{10}$

Using the scheme illustrated above (and the natural representation $\sigma =x_1$), we rewrite the Molien function for the irreducible representations in (9) in a form which is relevant for the computations of the generators of the invariants in $V_k\otimes \mathbb {C}[X,Y]$ (see Tables 7, 8, 9). The choice of the powers in the denominators is determined by the existence of invariants at those degrees. These invariants are called the *primary invariants*, while the ones corresponding to the terms in the numerator are called the *secondary invariants*.

Consider $\mathbb {T}^\flat$ primary invariants at degree six and eight, so that $M((\mathbb {T}_k\otimes \mathbb {C}[X,Y])^{\mathbb {T}^\flat },t)=\frac{N}{(1-t^6)(1-t^8)}$. The numerators *N* are then given in Table 7.

Similarly, considering $\mathbb {O}^\flat$ and $\mathbb {Y}^\flat$ primary invariants at degree eight and twelve, and twelve and twenty, respectively, one obtains Molien functions $M((\mathbb {O}_k\otimes \mathbb {C}[X,Y])^{\mathbb {O}^\flat },t)$ and $M((\mathbb {Y}_k\otimes \mathbb {C}[X,Y])^{\mathbb {Y}^\flat },t)$—see Tables 8 and 9 for the respective numerators.

**Table 8:** Molien functions of the irreducible representations: $M((\mathbb {O}_k\otimes \mathbb {C}[X,Y])^{\mathbb {O}^\flat },t)$

irrep	Dynkin	dim	Molien function numerator *N*
$\mathbb {O}_1$	$x_0$	1	$1+t^{18}$
$\mathbb {O}_2$	*y*	1	$t^6+t^{12}$
$$\mathbb {O}_3$$	*z*	2	$$ t^4+t^8 +t^{10}+t^{14}$$
$\underline{\mathbb {O}}_4^\flat$	$x_1$	2	$t+t^{7}+t^{11}+t^{17}$
$$\mathbb {O}_5^\flat $$	$$x_1\otimes y$$	2	$$ t^5+t^{7}+t^{11}+t^{13}$$
$\mathbb {O}_6$	$x_2\otimes y$	3	$t^4+t^6+t^8+t^{10}+t^{12}+t^{14}$
$\mathbb {O}_7$	$x_2$	3	$t^{2}+t^6+t^8+t^{10}+t^{12}+t^{16}$
$$\mathbb {O}_8^\flat $$	$$x_3$$	4	$$ t^3+t^{5}+t^7+2t^9+t^{11}+t^{13}+t^{15}$$

**Table 9:** Molien functions of the irreducible representations: $M((\mathbb {Y}_k\otimes \mathbb {C}[X,Y])^{\mathbb {Y}^\flat },t)$

irrep	Dynkin	dim	Molien function numerator *N*
$\mathbb {Y}_1$	$x_0$	1	$1+t^{30}$
$\underline{\mathbb {Y}}_2^\flat$	$x_1$	2	$t+t^{11}+t^{19}+t^{29}$
$\mathbb {Y}_3^\flat$	*y*	2	$t^7+t^{13}+t^{17}+t^{23}$
$\mathbb {Y}_4$	*z*	3	$t^6+t^{10}+t^{14}+t^{16}+t^{20}+t^{24}$
$$\mathbb {Y}_5$$	$$x_2$$	3	$$ t^2+t^{10}+t^{12}+t^{18}+t^{20}+t^{28}$$
$$\mathbb {Y}_6$$	$$x_1 \otimes y$$	4	$$ t^6+t^8+t^{12}+t^{14}+t^{16}+t^{18}+t^{22}+t^{24}$$
$$\mathbb {Y}_7^\flat $$	$$x_3$$	4	$$ t^3+t^9+t^{11}+t^{13}+t^{17}+t^{19}+t^{21}+t^{27}$$
$$\mathbb {Y}_8$$	$$x_4$$	5	$$ t^4+t^8+t^{10}+t^{12}+t^{14}+t^{16}+t^{18}+t^{20}+t^{22}+t^{26}$$
$\mathbb {Y}_9^\flat$	$x_5$	6	$t^5+t^7+t^9+t^{11}+t^{13}+2t^{15}+t^{17}+t^{19}+t^{21}+t^{23}+t^{25}$

If one would like to compute the Molien function of a reducible representation, this is done by adding the Molien functions of the irreducible components with the corresponding multiplicities.

## Invariant Matrices

A brute-force computational approach towards invariant matrices consists in taking a general element in ${{\mathfrak {g}}}(V)\otimes \mathbb {C}[X,Y]$ of the degree dictated by the Molien function of ${{\mathfrak {g}}}(V)$, and average over the group $G^\flat$. The Molien function of ${{\mathfrak {g}}}(V)$ can be computed from the Molien functions of Tables 7, 8, and 9 and the decompositions in Tables 4, 5, and 6, using the additive property of the Molien function. This approach is, however, not very effective computationally, as, for example, it would imply averaging an element in ${{\mathfrak {sl}}}(\mathbb {Y}^\flat _9)\otimes \mathbb {C}_{28}[X,Y]$ (that is, of *X*, *Y*-degree twenty-eight).

Instead, we use the method of classical invariant theory to compute higher-order invariants by transvection, starting from lower degree ${{\mathfrak {g}}}(V)$-*ground forms*, where *V* is an irreducible $G^\flat$-representation. Hence, this reduces the problem to finding lower degree ${{\mathfrak {g}}}(V)$-ground forms. Moreover, transvection only involves multiplication and differentiation with respect to *X* and *Y*; thus, it is computationally very effective and easy to implement.

In order to systematically find the lower degrees ${{\mathfrak {g}}}(V)$-ground forms, one can use of the decomposition of ${{\mathfrak {g}}}(V)$ into irreducible representations. The degree of the ground form is the lowest degree in the Taylor expansion at $t=0$ of the Molien function (see Sect. 2.3) of the irreducible component in the decomposition (see Sect. 2.2.5); e.g. the degree for the $\mathbb {Y}_8$-ground form is four, see Tables 6 and 9; such ground form will be notated by ${{\mathfrak {A}}}_8^4$, where the upper index indicates the degree, while the lower one the corresponding *V*. The explicit projection on the irreducible components will be given in the next section.

### Fourier Transform

Let *W* be a finite-dimensional representation of a finite group $G^\flat$, and let $\{w_i\,|\,i=1,\ldots ,\dim W\}$ be a basis of *W*. Then, *W* can be decomposed as a direct sum of irreducible representations of $G^\flat$ as follows.

Let *V* be such an irreducible $G^\flat$-representation, and let $\{v^i\,|\,i=1,\ldots ,\dim V^*\}$ be a basis of $V^*$. Let $\langle W, V\rangle$ be the multiplicity of *V* in *W* (that is, *V* occurs as a direct summand in *W* $\langle W, V\rangle$ times) and consider the space of invariants

$$\begin{aligned} (W\otimes V^{*})^{G^\flat } =\left\{ \eta ^{k}\;|\; k=1,\ldots ,\langle W, V\rangle \right\} ,\quad \eta ^{k}=\sum _{i,j}\eta ^{k}_{i,j} \,w_i \otimes v^j. \end{aligned}$$

The $\eta ^k$ are traces of the basis of $V^*$ and its canonical dual basis, a basis for *V*. From the expression for $\eta ^k$, we find $\langle W,V\rangle$ *V*-bases $\{v_j^k=\sum _{i}\eta _{i,j}^k w_i\,|\,j=1,\ldots ,\dim V\}$, $k=1,\ldots ,\langle W, V\rangle$.

In practice, we take a general element $\sum _{i,j} \zeta _{i,j} \,w_i \otimes v^j$ in $W\otimes V^*$ and require this element to be invariant under the action of the generators of $G^\flat$ to obtain elements $\eta ^k=\sum _{i,j}\eta ^{k}_{i,j} \,w_i \otimes v^j$.

If we now do the same construction for $U\otimes V$, we find $V^*$-bases in *U*. Taking the trace with each *V*-basis in *W* results in $\langle W, V\rangle \langle U, V^*\rangle$ linearly independent elements of $(W\otimes U)^{G^\flat }$. The space spanned by these elements will be denoted by $(W\otimes U)_V^{G^\flat }$. We have

$$\begin{aligned} (W\otimes U)^{G^\flat }=\bigoplus _{V\in \text {Irr}G^\flat }(W\otimes U)_V^{G^\flat } \end{aligned}$$

We return to the original problem of finding invariant matrices of degree *d* in ${{\mathfrak {sl}}}(V)\otimes {\mathbb {k}}[X,Y]$. To this end, we apply the above construction to the $G^\flat$-representations ${{\mathfrak {sl}}}(V)$ and ${\mathbb {k}}_d[X,Y]$ and obtain $({{\mathfrak {sl}}}(V)\otimes {\mathbb {k}}_d[X,Y])_{V'}^{G^\flat }$, with $V'\in \text {Irr}(G^\flat )$.

### Transvectants

In classical invariant theory, the basic computational tool is the *transvectant*: given any two invariants (in the context of invariant theory these are called covariants), it is possible to construct a number of (possibly new) invariants by computing transvectants. As a simple example, consider two linear forms $$aY+bX$$, $$cY+dX$$; their first transvectant is the determinant of the coefficients, i.e. $$ad-cb$$. A transformation on (*X*, *Y*) induces a transformation on (*a*, *b*) such that $$aY+bX$$ remains constant, and similarly for (*c*, *d*). Then, $$ad-cb$$ is invariant under the joint induced transformations on (*a*, *b*) and (*c*, *d*). Similarly, the discriminant $$ a_0 a_2-a^{2}_{1}$$ of a quadratic form $$a_0 Y^2+2 a_1 X Y+a_2 X^2$$ is the second transvectant of the quadratic form with itself. While the transvectant language has been superseded by more general constructions, working for all finite-dimensional Lie algebras, and sounds rather old-fashioned to present day algebraists, it is still a very effective computational tool when it can be applied and it is easy to program. The only assumption one makes is that the group acts linearly and faithfully on $$\mathbb {C}^2$$, that the group elements are represented by matrices in SL$$_{2}(\mathbb {C})$$, as it is indeed the case for the natural representation $$\sigma $$ (see Definition 2.3). If one would like to replace $$\mathbb {C}^2$$ by a higher-dimensional space, the transvectant mechanism is no longer available, but while the transvectant technique is very efficient, the results in this paper could also have been obtained without transvectants, e.g. using group averaging as mentioned at the beginning of Sect. 3.

In this section, we will adapt the idea of transvection to compute invariant Lie algebras. We start from the classical work by Klein about automorphic functions and generalise it to the context of automorphic algebras. To do so, we need first to recall some definitions and facts about transvectants and generalise some of the concepts to the present set-up.

Recall the Definition 2.1 of relative invariant; in the literature, relative invariants are also called semi-invariants or covariants.

#### **Definition 3.1**

*(Polynomial ground form*) A **polynomial ground form** is a relative invariant polynomial ${{\mathfrak {a}}}$ of minimal degree. The divisor of zeros of such a polynomial is an exceptional (or degenerate) *G*-orbit of minimal order.

#### **Definition 3.2**

(*Ground form*) A **ground form** is an invariant ${{\mathfrak {A}}}\in {\mathcal {V}}$ of minimal degree, where ${\mathcal {V}}$ is a *G*-module and a ${\mathbb {k}}[X,Y]$-module.

The computations of polynomial ground forms for the $\mathbb {T}\mathbb {O}\mathbb {Y}$ groups can be found, for instance, in [6], [22, II.6] and [14].

#### **Definition 3.3**

(*Transvectant*) Let ${\mathcal {V}}$ and ${\mathcal {W}}$ be *G*-modules and ${\mathbb {k}}[X,Y]$-modules. Let $\phi \in {\mathcal {V}}_G$ and $\phi _{k,l}=\frac{\partial ^{k+l}\phi }{\partial X^k\partial Y^l}$; we define the *k*th-**transvectant** of $\phi$ with $\psi \in {\mathcal {W}}_G$

$$\begin{aligned} {{\mathfrak {F}}}=(\phi ,\psi )^k =\sum _{i=0}^k (-1)^i\left( {\begin{array}{c}k\\ i\end{array}}\right) \phi _{i,k-i}\otimes \psi _{k-i,i}\in ({\mathcal {V}}\otimes {\mathcal {W}})_G. \end{aligned}$$

#### **Lemma 3.1**

Let $\phi \in {\mathcal {V}}_G$ and $\psi \in {\mathcal {W}}_G$; the transvectant transforms as

$$\begin{aligned} g(\phi ,\psi )^k =(g\phi ,g\psi )^k , \quad g\in G. \end{aligned}$$

This implies that $(\phi ,\psi )^k\in ({\mathcal {V}}\otimes {\mathcal {W}})_G$, and if $\phi$ and $\psi$ are invariant, so is $(\phi ,\psi )^k$.

#### **Corollary 3.1**

Let ${{\mathfrak {A}}}\in {\mathcal {V}}$ be a ground form and $\bar{{{\mathfrak {a}}}}$ an invariant polynomial. Then $(\bar{{{\mathfrak {a}}}},{{\mathfrak {A}}})^l \in {\mathcal {V}}^G$.

#### **Corollary 3.2**

Let $\phi \in ({\mathcal {V}}\otimes V)^G$ and $\psi \in (V^*\otimes {\mathbb {k}}[X,Y])^G$. Let ${{\mathfrak {A}}}=\mathrm {Trace\ } \phi \otimes \psi \in {\mathcal {V}}^G$ be an invariant form, Then $(\bar{{{\mathfrak {a}}}},{{\mathfrak {A}}})^l=\mathrm {Trace\ } \phi \otimes (\bar{{{\mathfrak {a}}}},\psi )^l\in {\mathcal {V}}^G$, with $\bar{{{\mathfrak {a}}}}$ a polynomial invariant.

This justifies the way we compute a sequence of invariants from a ground form using the Molien function of the irreducible representation *V* (see Sect. 3).

#### *Example 3.1*

The polynomial ground forms for $\mathbb {T},\mathbb {O}$ and $\mathbb {Y}$, in the bases given by (4), (5) and (6), respectively, are:

$$\begin{aligned} {{\mathfrak {a}}}_{4,1}= & {} Y (X - 1/2Y ) (X - 1/2\omega _3 Y) \left( X-1/2\omega _3^2Y \right) \\ {{\mathfrak {a}}}_{6,1}= & {} \left( X + \omega _{24}^5 Y + \omega _{24}^7 Y\right) \left( X + \omega _{24} Y + \omega _{24}^3 Y - \omega _{24}^5 Y - \omega _{24}^7 Y \right) \\&\times \left( X -\omega _{24} Y - \omega _{24}^3 Y\right) \left( X - \omega _{24} Y + \omega _{24}^3 Y - \omega _{24}^7 Y\right) \\&\times \left( X - \omega _{24}^3 Y + \omega _{24}^5 Y \right) \left( X + \omega _{24} Y - \omega _{24}^5 Y + \omega _{24}^7 Y \right) \\ {{\mathfrak {a}}}_{12}= & {} (X) (Y) \left( X - Y + \omega _{5}^2 Y + \omega _{5}^3 Y\right) \left( X + \omega _{5}^3 Y \right) \left( X + \omega _{5}^4 Y \right) \left( X + \omega _{5}^2 Y\right) \\&\times \left( X+ Y + \omega _{5} Y - \omega _{5}^3 Y \right) \left( X + \omega _{5} Y\right) \left( X - \omega _{5} Y - 2 \omega _{5}^2 Y - \omega _{5}^3 Y \right) \\&\times \left( X - Y - 2 \omega _{5} Y - \omega _{5}^2 Y\right) \left( X+ Y + 2 \omega _{5} Y + 2 \omega _{5}^2 Y + \omega _{5}^3 Y\right) (X+ Y ) \end{aligned}$$

The subindex of ${{\mathfrak {a}}}_{i,j}$ is determined as follows: *i* is the *X*, *Y*-degree and *j* identifies the element in the group of one-dimensional characters describing how ${{\mathfrak {a}}}_{i,j}$ transforms. For example, the one-dimensional characters of $\mathbb {T}$ constitute the group $\mathbb {Z}/3=\{0,1,2\}$ by identifying $\mathbb {T}_{j+1}$ with $j\in \mathbb {Z}/3$. In ${{\mathfrak {a}}}_{12}$, the second grading is trivial, so it is omitted (see also Examples 3.3–3.5).

Recall Definition 2.1; let ${\mathbb {k}}[X,Y]_G$ denote the ring of relative invariants and ${\mathbb {k}}[X,Y]^G$ the ring of invariants.

#### *Example 3.2*

(Classical Invariant Theory) Let $V=W={\mathbb {k}}[X,Y]_G$ and replace in the Definition 3.3 the tensor product by the ordinary product of polynomials. Then ${{\mathfrak {F}}}\in {\mathbb {k}}[X,Y]_G$. Let ${{\mathfrak {a}}}$ be the lowest degree relative invariant, then it follows from the classical theory that if *G* is either $\mathbb {T}$, $\mathbb {O}$ or $\mathbb {Y}$ the classical relative invariants [13, 14] are given by

$$\begin{aligned} {{\mathfrak {a}}},\quad {{\mathfrak {b}}}=({{\mathfrak {a}}},{{\mathfrak {a}}})^{2},\quad {{\mathfrak {c}}}=({{\mathfrak {a}}},{{\mathfrak {b}}})^{1}. \end{aligned}$$

**Table 10:** Degrees of the classical relative invariants of $\mathbb {T}, \mathbb {O}, \mathbb {Y}$

*G*	$\deg _{{\mathfrak {a}}}$	$\deg _{{\mathfrak {b}}}=2\deg _{{\mathfrak {a}}}-4$	$\deg _{{\mathfrak {c}}}=3\deg _{{\mathfrak {a}}}-6$
$\mathbb {T}$	4	4	6
$\mathbb {O}$	6	8	12
$\mathbb {Y}$	12	20	30

If one denotes the degree of a form ${{\mathfrak {a}}}$ by $\deg _{{\mathfrak {a}}}$, it follows that (see Table 10)

$$\begin{aligned} \deg _{{\mathfrak {b}}}=2\,\deg _{{\mathfrak {a}}}-4,\quad \deg _{{\mathfrak {c}}}=3\,\deg _{{\mathfrak {a}}}-6. \end{aligned}$$

The degree of ${{\mathfrak {b}}}$ is the number of faces of the Platonic solid and determines its name. We observe that $\deg _{{\mathfrak {a}}}-\deg _{{\mathfrak {c}}}+\deg _{{\mathfrak {b}}}=2$, the Euler characteristic, and that $\deg _{{\mathfrak {a}}}+\deg _{{\mathfrak {b}}}+\deg _{{\mathfrak {c}}}=|G|+2$.

The next examples illustrate how the Molien series information is combined with the concept of transvectant to construct a basis for the relative invariants. We write ${\mathbb {k}}[V]={\mathbb {k}}[X,Y]$ when $\{X,Y\}$ is a basis for the dual of a natural representation *V*.

#### *Example 3.3*

(Tetrahedral group $\mathbb {T}$) The ring generated by the relative invariants is determined as follows. From GAP, we obtain the Molien function

$$\begin{aligned} M\left( {\mathbb {k}}[\mathbb {T}_4^\flat ]_{\mathbb {T}^\flat },t \right) =\frac{1+2t^4+2t^8+t^{12}}{(1-t^6)(1-t^8)} =\frac{1-t^{12}}{(1-t^4)^2(1-t^6)} =\frac{1+t^{6}}{(1-t^4)^2}. \end{aligned}$$

To find the ground form ${{\mathfrak {a}}}_{4,1}$, we look in ${\mathbb {T}_2}\otimes {\mathbb {k}}_4[\mathbb {T}_4^\flat ]$. Then ${{\mathfrak {b}}}_{4,2}=({{\mathfrak {a}}}_{4,1},{{\mathfrak {a}}}_{4,1})^2\in {\mathbb {k}}_4[\mathbb {T}_4^\flat ]^{\mathbb {T}_3}$ and ${{\mathfrak {c}}}_{6,0}=({{\mathfrak {a}}}_{4,1},{{\mathfrak {b}}}_{4,2})^1\in {\mathbb {k}}_6[\mathbb {T}_4^\flat ]^{\mathbb {T}^\flat }$, in analogy with classical invariant theory. This follows from Table 10. Thus, one finds that

$$\begin{aligned} {\mathbb {k}}[\mathbb {T}_4^\flat ]_{\mathbb {T}^\flat }={\mathbb {k}}[{{\mathfrak {a}}}_{4,1},{{\mathfrak {b}}}_{4,2}](1\oplus {{\mathfrak {c}}}_{6,0}) \end{aligned}$$

where

$$\begin{aligned} {{\mathfrak {a}}}_{4,1}= & {} Y^4 - 8X^3Y\\ {{\mathfrak {b}}}_{4,2}= & {} - 1152XY^3 - 1152X^4 \end{aligned}$$

and

$$\begin{aligned} {{\mathfrak {c}}}_{6,0}=- 4608Y^6 - 92160X^3Y^3 + 36864X^6 \end{aligned}$$

in the basis given by (4). One expects from the Molien function a relation at degree 12 of the form

$$\begin{aligned} {{\mathfrak {a}}}_{4,1}^3+C_{{\mathfrak {a}}}^{{\mathfrak {b}}}{{\mathfrak {b}}}_{4,2}^3+C_{{\mathfrak {a}}}^{{\mathfrak {c}}} {{\mathfrak {c}}}_{6,0}^2=0,\quad C_{{\mathfrak {a}}}^{{\mathfrak {b}}},\,C_{{\mathfrak {a}}}^{{\mathfrak {c}}}\in {\mathbb {k}}^*\end{aligned}$$

and one finds $C_{{\mathfrak {a}}}^{{\mathfrak {b}}}= - 1/23887872$ and $C_{{\mathfrak {a}}}^{{\mathfrak {a}}}= - 1/21233664$. The Molien function of the invariants is given by

$$\begin{aligned} M\left( {\mathbb {k}}[\mathbb {T}_4^\flat ]^{\mathbb {T}^\flat },t\right) =\frac{1+t^{12}}{(1-t^6)(1-t^8)}. \end{aligned}$$

Thus, the invariants corresponding to these terms are ${{\mathfrak {c}}}_{6,0}\equiv \bar{{{\mathfrak {a}}}}_{6}$ for $t^6$, ${{\mathfrak {a}}}_{4,1}{{\mathfrak {b}}}_{4,2}\equiv \bar{{{\mathfrak {b}}}}_{8}$ for $t^{8}$ and ${{\mathfrak {a}}}_{4,1}^3\equiv \bar{{{\mathfrak {c}}}}_{12}$ for $t^{12}$ (or equivalently ${{\mathfrak {b}}}_{4,2}^3$). Hence, the ring of invariants can be written as

$$\begin{aligned} {\mathbb {k}}[\mathbb {T}_4^\flat ]^{\mathbb {T}^\flat }={\mathbb {k}}[\bar{{{\mathfrak {a}}}}_{6},\bar{{{\mathfrak {b}}}}_{8}](1\oplus \bar{{{\mathfrak {c}}}}_{12}). \end{aligned}$$

#### *Example 3.4*

(Octahedral group $\mathbb {O}$) Similarly, the ring generated by the $\mathbb {O}$-relative invariants is determined as follows. From GAP, we obtain the Molien function

$$\begin{aligned} M\left( {\mathbb {k}}[\mathbb {O}_4^\flat ]_{\mathbb {O}^\flat },t\right) =\frac{1+t^6+t^{12}+t^{18}}{(1-t^8)(1-t^{12})} =\frac{1+t^{12}}{(1-t^6)(1-t^8)} \end{aligned}$$

and the individual generating function for $\mathbb {O}_2$ is

$$\begin{aligned} M\left( {\mathbb {k}}[\mathbb {O}_4^\flat ]^{\mathbb {O}_2},t\right) =\frac{t^6+t^{12}}{(1-t^8)(1-t^{12})} \end{aligned}$$

and for $\mathbb {O}_1$ is

$$\begin{aligned} M\left( {\mathbb {k}}[\mathbb {O}_4^\flat ]^{\mathbb {O}^\flat },t\right) =\frac{1+t^{18}}{(1-t^8)(1-t^{12})}. \end{aligned}$$

To find the basic covariant ${{\mathfrak {a}}}_{6,1}$, we look in ${\mathbb {k}}_6[\mathbb {O}_4^\flat ]^{\mathbb {O}_2}$. Then, ${{\mathfrak {b}}}_{8,0}=({{\mathfrak {a}}}_{6,1},{{\mathfrak {a}}}_{6,1})^2\in {\mathbb {k}}_8[\mathbb {O}_4^\flat ]^{\mathbb {O}^\flat }$ and ${{\mathfrak {c}}}_{12,1}=({{\mathfrak {a}}}_{6,1},{{\mathfrak {b}}}_{8,0})^1\in {\mathbb {k}}_{12}[\mathbb {O}_4^\flat ]^{\mathbb {O}_2}$. Thus one finds that

$$\begin{aligned} {\mathbb {k}}[\mathbb {O}_4^\flat ]_{\mathbb {O}^\flat }={\mathbb {k}}[{{\mathfrak {a}}}_{6,1},{{\mathfrak {b}}}_{8,0}](1\oplus {{\mathfrak {c}}}_{12,1}). \end{aligned}$$

We identify the terms in the Molien function for $\mathbb {O}_1$: the $t^8$ is $\bar{{{\mathfrak {a}}}}_{8}={{\mathfrak {b}}}_{8,0}$, the $t^{12}$-term is $\bar{{{\mathfrak {b}}}}_{12}={{\mathfrak {a}}}_{6,1}^2$ and the $t^{18}$-term is $\bar{{{\mathfrak {c}}}}_{18}={{\mathfrak {a}}}_{6,1}{{\mathfrak {c}}}_{12,1}$. We identify the terms in the numerator of the $\mathbb {O}_2$-Molien function as follows. The $t^6$ term is ${{\mathfrak {a}}}_{6,1}$, and the $t^{12}$ term is ${{\mathfrak {c}}}_{12,1}$.

One can check that the relative invariants satisfy a relation of the form

$$\begin{aligned} {{\mathfrak {a}}}_{6,1}^4+C_{{\mathfrak {a}}}^{{\mathfrak {b}}} {{\mathfrak {b}}}_{8,0}^3+C_{{\mathfrak {a}}}^{{\mathfrak {c}}} {{\mathfrak {c}}}_{12,1}^2=0. \end{aligned}$$

It follows that the invariants have the following relation

$$\begin{aligned} C_{{\mathfrak {a}}}^{{\mathfrak {b}}} \bar{{{\mathfrak {a}}}}_{8}^3\bar{{{\mathfrak {b}}}}_{12}+\bar{{{\mathfrak {b}}}}_{12}^3+C_{{\mathfrak {a}}}^{{\mathfrak {c}}} \bar{{{\mathfrak {c}}}}_{18}^2=0 \end{aligned}$$

and that the ring of invariants can be written as

$$\begin{aligned} {\mathbb {k}}[\mathbb {O}_4^\flat ]^{\mathbb {O}^\flat }={\mathbb {k}}[\bar{{{\mathfrak {a}}}}_{8},\bar{{{\mathfrak {b}}}}_{12}](1\oplus \bar{{{\mathfrak {c}}}}_{18}). \end{aligned}$$

#### *Example 3.5*

(Icosahedral group $\mathbb {Y}$) The Molien function of the invariants is

$$\begin{aligned} M\left( {\mathbb {k}}[\mathbb {Y}_2^\flat ]^{\mathbb {Y}^\flat },t\right) =\frac{1+t^{30}}{(1-t^{12})(1-t^{20})}. \end{aligned}$$

The invariants are ${{\mathfrak {a}}}_{12}$, ${{\mathfrak {b}}}_{20}=({{\mathfrak {a}}}_{12},{{\mathfrak {a}}}_{12})^2$ and ${{\mathfrak {c}}}_{30}=({{\mathfrak {a}}}_{12},{{\mathfrak {b}}}_{20})^1$, and they satisfy the following relation

$$\begin{aligned} {{\mathfrak {a}}}_{12}^5+C_{{\mathfrak {a}}}^{{\mathfrak {b}}} {{\mathfrak {b}}}_{20}^3+C_{{\mathfrak {a}}}^{{\mathfrak {c}}} {{\mathfrak {c}}}_{30}^2=0. \end{aligned}$$

The ring of invariants can be written as

$$\begin{aligned} {\mathbb {k}}[\mathbb {Y}_2^\flat ]^{\mathbb {Y}^\flat }={\mathbb {k}}[{{\mathfrak {a}}}_{12},{{\mathfrak {b}}}_{20}](1\oplus {{\mathfrak {c}}}_{30}). \end{aligned}$$

### ${\mathbb {T}}{\mathbb {O}}{\mathbb {Y}}$-Invariant Matrices

Our goal is to determine the structure of the Lie algebra of invariant matrices. Once the ground forms are computed, the other degrees can be realised by taking appropriate transvectants with the relative invariants. The choice of transvectants is completely independent of the dimension we are working in; thus, the construction is completely uniform.

We observe in the first place that it is possible to predict that the number of generators of $(V\otimes {\mathbb {k}}[X,Y])^{G^\flat }$ is twice the dimension of *V*. This follows from the following Lemma, a modification of a method by Stanley [37].

#### **Lemma 3.2**

Let $G^\flat$ be a finite subgroup of $\text {SL}(2,\mathbb {C})$, and let *V* be its irreducible representation with character $\chi$. The space of invariants $(V\otimes {\mathbb {k}}[X,Y])^{G^\flat }$ is a Cohen–Macaulay module of Krull dimension 2. Say

$$\begin{aligned} (V\otimes {\mathbb {k}}[X,Y])^{G^\flat }=\bigoplus _{i=1}^{k_\chi }{\mathbb {k}}[\bar{{{\mathfrak {a}}}},\bar{{{\mathfrak {b}}}}]\rho _i \end{aligned}$$

and set $e_i=\deg \rho _i$. Then,

10 $$\begin{aligned} k_\chi |G^\flat |= & {} \deg _{\bar{{{\mathfrak {a}}}}} \deg _{\bar{{{\mathfrak {b}}}}} \chi (1)\end{aligned}$$

11 $$\begin{aligned} \frac{2}{k_\chi }\sum _{i=1}^{k_\chi }e_i= & {} \deg _{\bar{{{\mathfrak {a}}}}} +\deg _{\bar{{{\mathfrak {b}}}}} -2. \end{aligned}$$

#### *Proof*

The two equations follow from the first two coefficients, *A* and *B*, of the Laurent expansion around $t=1$ of the Molien series

$$\begin{aligned} M\left( (V\otimes {\mathbb {k}}[X,Y])^{G^\flat },t\right) =\frac{A}{(1-t)^2}+\frac{B}{1-t}+{\mathcal {O}}(1). \end{aligned}$$

We have two ways to express this series, namely by Molien’s theorem and by the expression of $(V\otimes {\mathbb {k}}[X,Y])^{G^\flat }$ as a Cohen–Macaulay module.

First Molien’s theorem: $P(V\otimes {\mathbb {k}}[X,Y])^{G^\flat },t)=\frac{1}{|G^\flat |}\sum _{g\in G^\flat }\frac{\overline{\chi (g)}}{\det (1-t\sigma (g))}$. We see that the only contribution to the term of order $(1-t)^{-2}$ in the Laurent expansion comes from the identity element $g=1$, so $A=\frac{\chi (1)}{|G^\flat |}.$ The terms $\frac{\overline{\chi (g)}}{\det (1-t\sigma (g))}$ that contribute to the coefficient of $(1-t)^{-1}$ in the Laurent expansion come from elements $\sigma (g)$ that have precisely one eigenvalue equal to 1. However, since $\det \sigma (g)=1$ there are no such elements: $B=0$.

On the other hand, we notice that

$$\begin{aligned} P\left( \bigoplus _{i=1}^{k_\chi }{\mathbb {k}}[\bar{{{\mathfrak {a}}}},\bar{{{\mathfrak {b}}}}]\rho _i,t\right) =\frac{\sum _{i=1}^{k_\chi }t^{e_i}}{(1-t^{\deg _{\bar{{{\mathfrak {a}}}}}})(1-t^{\deg _{\bar{{{\mathfrak {b}}}}}})} \end{aligned}$$

and the first two coefficients of the Laurent expansion around $t=1$ are $A=\frac{k_\chi }{\deg _{\bar{{{\mathfrak {a}}}}}\deg _{\bar{{{\mathfrak {b}}}}}}$ and $B=\frac{k_\chi }{2 \deg _{\bar{{{\mathfrak {a}}}}}\deg _{\bar{{{\mathfrak {b}}}}}}(\deg _{\bar{{{\mathfrak {a}}}}}-1)+\frac{k_\chi }{2\deg _{\bar{{{\mathfrak {a}}}}}\deg _{\bar{{{\mathfrak {b}}}}}}(\deg _{\bar{{{\mathfrak {b}}}}}-1)-\frac{1}{\deg _{\bar{{{\mathfrak {a}}}}}\deg _{\bar{{{\mathfrak {b}}}}}}\sum _{i=1}^{k_\chi }e_i$. The result follows. $\square$

In Sect. 4, we then repeat the procedure of Sect. 3, with a slight variation, to produce a basis for relative invariant vectors.

In the following sections, we compute a basis for |*G*|-homogeneous *G*-invariant matrices; this is a minimal generating set for the module of *G*-invariant matrices (over the primary invariants ${{\mathfrak {a}}}^{d_G}$ and ${{\mathfrak {b}}}^3$) whose homogeneous elements have degree divisible |*G*|. This will be enough to construct a minimal generating set for the Automorphic Lie Algebra (see [17, 19]).

#### Tetrahedral Group Invariant Matrices

From Table 4, it follows that ${{\mathfrak {g}}}(V)$ splits into a direct sum of $\mathbb {T}_i, i=2,3,7$. We then consider $(\mathbb {T}_i\otimes {\mathbb {k}}_{12}[\mathbb {T}_4^\flat ])^{\mathbb {T}^\flat }$, as it is sufficient to consider entries of degree equal to the order of the group $|\mathbb {T}|$ (see [17, 19]).

The ground forms and transvectants are listed in Table 11. Notice that the degrees in column Molien and Multiplier add up to the order of the group.

**Table 11:** Generators of $\mathbb {T}$-invariant matrices of degree $|\mathbb {T}|$

irrep	Molien	Ground form	Invariant matrix	Multiplier
$\mathbb {T}_1$	1	${{\mathfrak {A}}}_1^0$	${{\mathfrak {M}}}_1^0={{\mathfrak {A}}}_1^0$	$\bar{{{\mathfrak {a}}}}_{6}^2$
$\mathbb {T}_2$	$t^4$	${{\mathfrak {A}}}_2^4$	${{\mathfrak {M}}}_2^4={{\mathfrak {A}}}_2^4$	$\bar{{{\mathfrak {b}}}}_{8}$
$\mathbb {T}_3$	$t^4$	${{\mathfrak {A}}}_3^4$	${{\mathfrak {M}}}_3^4={{\mathfrak {A}}}_3^4$	$\bar{{{\mathfrak {b}}}}_{8}$
$\mathbb {T}_7$	$t^4$	${{\mathfrak {A}}}_7^2$	${{\mathfrak {M}}}_7^4=\left( \bar{{{\mathfrak {a}}}}_{6},{{\mathfrak {A}}}_7^2\right) ^2$	$\bar{{{\mathfrak {b}}}}_{8}$
	$t^6$		${{\mathfrak {M}}}_7^{6}=\left( \bar{{{\mathfrak {a}}}}_{6},{{\mathfrak {A}}}_7^{2}\right) ^1$	$\bar{{{\mathfrak {a}}}}_{6}$
	$t^6$		${{\mathfrak {N}}}_7^{6}=\left( \bar{{{\mathfrak {b}}}}_{8},{{\mathfrak {A}}}_7^{2}\right) ^2$	$\bar{{{\mathfrak {a}}}}_{6}$

Table 11 is constructed by considering first the decomposition in Table 4; one observes that the only representations playing a role are $\mathbb {T}_2$, $\mathbb {T}_3$ and $\mathbb {T}_7$, so they are listed in the first column of Table 11. The trivial representation $\mathbb {T}_1$ is added for future reference. Next one considers the numerators of their corresponding Molien functions (see Table 7): the lowest order terms ($t^4$, $t^4$ and $t^2$), computed using the technique of Sect. 3.1, are the ground forms ${{\mathfrak {A}}}_2^4$, ${{\mathfrak {A}}}_3^4$ and ${{\mathfrak {A}}}_7^2$ in the third column, where the upper index denotes the degree in *X* and *Y* and the lower index refers to the irreducible representation (see the first column). The fourth column contains the invariant matrices; the last three entries correspond to $t^4$ and $2t^6$ in the $\mathbb {T}_7$-row are obtained by taking the first transvectant with the primary invariants $\bar{{{\mathfrak {b}}}}_{8}$, $\bar{{{\mathfrak {a}}}}_{6}$. It is worth noticing that not all terms in the numerator of the Molien function are present. This is due to the fact that not all invariant matrices can be made |*G*|-homogeneous: for instance, looking at Table 7 for $\mathbb {T}_2$, we observe that the $t^{8}$ term is missing; indeed, in this case one would need to solve the linear diophantine equation $6n+8m+8=|\mathbb {T}|=12$, which has no solutions for *n* and *m* nonnegative integer. The last column of Table 11 illustrates that one can solve the diophantine equation for the terms in the second column; hence, a basis for $|\mathbb {T}|$-homogeneous $\mathbb {T}^\flat$-invariant matrices is given by the products of the elements in the last two columns.

##### *Example 3.6*

From Table 4, one has ${{\mathfrak {sl}}}_{2}(\mathbb {T}_5^\flat )\cong \mathbb {T}_7$. To find a concretisation of ${{\mathfrak {A}}}_7^2$, we consider an embedding $\vartheta ^{{{\mathfrak {sl}}}_{2}(\mathbb {T}_5^\flat )}$ of $\mathbb {T}_7$ into ${{\mathfrak {sl}}}_{2}(\mathbb {T}_5^\flat )$:

$$\begin{aligned} \vartheta ^{{{\mathfrak {sl}}}_{2}(\mathbb {T}_5^\flat )}({{\mathfrak {A}}}_7^2)= \begin{pmatrix} XY &{}\quad 1/2Y^2 \\ - 2X^2 &{}\quad - XY \end{pmatrix}. \end{aligned}$$

In the case of ${{\mathfrak {sl}}}_{3}(\mathbb {T}_7)\cong \mathbb {T}_2\oplus \mathbb {T}_3\oplus 2 \mathbb {T}_7$, one has two concretisations of the ground form ${{\mathfrak {A}}}_7^2$, namely $\vartheta _{1}^{{{\mathfrak {sl}}}_{3}(\mathbb {T}_7)}({{\mathfrak {A}}}_7^2)$ and $\vartheta _{2}^{{{\mathfrak {sl}}}_{3}(\mathbb {T}_7)}({{\mathfrak {A}}}_7^2)$, since the multiplicity of $\mathbb {T}_7$ in ${{\mathfrak {sl}}}_{3}(\mathbb {T}_7)$ is two.

##### *Example 3.7*

We compute a set of generators for ${{\mathfrak {sl}}}_3(\mathbb {T}_7)$, linearly independent over the ring ${\mathbb {k}}[\bar{{{\mathfrak {a}}}}_{6},\bar{{{\mathfrak {b}}}}_{8}]$ of primary invariants. We know that ${{\mathfrak {sl}}}_3(\mathbb {T}_7)\cong \mathbb {T}_2\oplus \mathbb {T}_3\oplus 2 \mathbb {T}_7$. Therefore, we have ground forms ${{\mathfrak {A}}}_2^4$, ${{\mathfrak {A}}}_3^4$ and ${{\mathfrak {A}}}_{7}^2$. Thus we compute the generators $\vartheta ^{{{\mathfrak {sl}}}_{3}(\mathbb {T}_7)}({{\mathfrak {M}}}_2^4)$, $\vartheta ^{{{\mathfrak {sl}}}_{3}(\mathbb {T}_7)}({{\mathfrak {M}}}_3^4)$, $\vartheta _{1}^{{{\mathfrak {sl}}}_{3}(\mathbb {T}_7)}({{\mathfrak {M}}}_{7}^4)$, $\vartheta _{1}^{{{\mathfrak {sl}}}_{3}(\mathbb {T}_7)}({{\mathfrak {M}}}_{7}^6)$, $\vartheta _{1}^{{{\mathfrak {sl}}}_{3}(\mathbb {T}_7)}({{\mathfrak {N}}}_{7}^6)$, $\vartheta _{2}^{{{\mathfrak {sl}}}_{3}(\mathbb {T}_7)}( {{\mathfrak {M}}}_{7}^4)$, $\vartheta _{2}^{{{\mathfrak {sl}}}_{3}(\mathbb {T}_7)}({{\mathfrak {M}}}_{7}^6)$, $\vartheta _{2}^{{{\mathfrak {sl}}}_{3}(\mathbb {T}_7)}( {{\mathfrak {N}}}_{7}^6)$. Once we have tested their independence, we know from the Molien function that they span the space $({{\mathfrak {sl}}}(\mathbb {T}_7)\otimes {\mathbb {k}}[\mathbb {T}_4^\flat ])^{\mathbb {T}^\flat }$.

#### Octahedral Group Invariant Matrices

Table 12 is computed in the same spirit as in the previous section; also in this case, not all terms in the numerator of the Molien function (see Table 8) correspond to invariant matrices, which can be made zero homogeneous; hence, they are not listed in Table 12.

**Table 12:** Generators of $\mathbb {O}$-invariant matrices of degree $|\mathbb {O}|$

irrep	Molien	Ground form	Invariant matrix	Multiplier
$\mathbb {O}_1$	1	${{\mathfrak {A}}}_1^0$	${{\mathfrak {M}}}_1^{0}={{\mathfrak {A}}}_1^0$	$\bar{{{\mathfrak {b}}}}_{12}^2$
$\mathbb {O}_2$	$t^{12}$	${{\mathfrak {A}}}_2^6$	${{\mathfrak {M}}}_2^{12}=\left( \bar{{{\mathfrak {a}}}}_{8},{{\mathfrak {A}}}_2^6\right) ^1$	$\bar{{{\mathfrak {b}}}}_{12}$
$\mathbb {O}_3$	$t^4$	${{\mathfrak {A}}}_3^4$	${{\mathfrak {M}}}_3^4={{\mathfrak {A}}}_3^4$	$\bar{{{\mathfrak {a}}}}_{8}\bar{{{\mathfrak {b}}}}_{12}$
	$t^8$		${{\mathfrak {M}}}_3^{8}=\left( \bar{{{\mathfrak {a}}}}_{8},{{\mathfrak {A}}}_3^{4}\right) ^2$	$\bar{{{\mathfrak {a}}}}_{8}^2$
$\mathbb {O}_6$	$t^4$	${{\mathfrak {A}}}_6^4$	${{\mathfrak {M}}}_6^4={{\mathfrak {A}}}_6^4$	$\bar{{{\mathfrak {a}}}}_{8}\bar{{{\mathfrak {b}}}}_{12}$
	$$ t^8$$		$${{\mathfrak {M}}}_6^{8}=\left( \bar{{{\mathfrak {a}}}}_{8},{{\mathfrak {A}}}_6^{4}\right) ^2$$	$$\bar{{{\mathfrak {a}}}}_{8}^2$$
	$$ t^{12}$$		$${{\mathfrak {M}}}_6^{12}=\left( \bar{{{\mathfrak {a}}}}_{8},{{\mathfrak {A}}}_6^{8}\right) ^2$$	$$\bar{{{\mathfrak {b}}}}_{12}$$
$$\mathbb {O}_7$$	$$ t^8$$	$${{\mathfrak {A}}}_7^2$$	$${{\mathfrak {M}}}_7^{8}=\left( \bar{{{\mathfrak {a}}}}_{8},{{\mathfrak {A}}}_7^{2}\right) ^1$$	$$\bar{{{\mathfrak {a}}}}_{8}^2$$
	$$ t^{12}$$		$${{\mathfrak {M}}}_7^{12}=\left( \bar{{{\mathfrak {a}}}}_{8},{{\mathfrak {M}}}_7^{8}\right) ^2$$	$$\bar{{{\mathfrak {b}}}}_{12}$$
	$$ t^{16}$$		$${{\mathfrak {M}}}_7^{16}=\left( \bar{{{\mathfrak {a}}}}_{8},{{\mathfrak {M}}}_7^{12}\right) ^2$$	$$\bar{{{\mathfrak {a}}}}_{8}$$

#### Icosahedral Group Invariant Matrices

The invariant matrices for $\mathbb {Y}^\flat$ are presented in Table 13; as before, not all terms in the numerator of the Molien function (see Table 9) correspond to invariant matrices, which can be made zero homogeneous; hence, they are not listed in Table 13.

**Table 13:** Generators of $\mathbb {Y}$-invariant matrices of degree $|\mathbb {Y}|$

irrep	Molien	Ground form	Invariant matrix	Multiplier
$\mathbb {Y}_1$	1	${{\mathfrak {A}}}_1^0$	${{\mathfrak {M}}}_1^{0}={{\mathfrak {A}}}_1^0$	${{\mathfrak {a}}}_{12}^5$
$\mathbb {Y}_4$	$t^{16}$	${{\mathfrak {A}}}_4^6$	${{\mathfrak {M}}}_4^{16}=\left( {{\mathfrak {a}}}_{12},{{\mathfrak {A}}}_4^6\right) ^1$	${{\mathfrak {a}}}_{12}^2{{\mathfrak {b}}}_{20}$
	$t^{20}$		${{\mathfrak {M}}}_4^{20}=\left( {{\mathfrak {a}}}_{12},{{\mathfrak {M}}}_4^{16}\right) ^4$	${{\mathfrak {b}}}_{20}^2$
	$t^{24}$		${{\mathfrak {M}}}_4^{24}=\left( {{\mathfrak {a}}}_{12},{{\mathfrak {M}}}_4^{20}\right) ^4$	${{\mathfrak {a}}}_{12}^3$
$\mathbb {Y}_5$	$t^{12}$	${{\mathfrak {A}}}_5^2$	${{\mathfrak {M}}}_5^{12}=\left( {{\mathfrak {a}}}_{12},{{\mathfrak {A}}}_5^2\right) ^1$	${{\mathfrak {a}}}_{12}^4$
	$$ t^{20}$$		$${{\mathfrak {M}}}_5^{20}=\left( {{\mathfrak {a}}}_{12},{{\mathfrak {M}}}_5^{12}\right) ^2$$	$${{\mathfrak {b}}}_{20}^2 $$
	$t^{28}$		${{\mathfrak {M}}}_5^{28}=\left( {{\mathfrak {a}}}_{12},{{\mathfrak {M}}}_5^{20}\right) ^2$	${{\mathfrak {a}}}_{12}{{\mathfrak {b}}}_{20}$
$\mathbb {Y}_6$	$t^8$	${{\mathfrak {A}}}_6^6$	${{\mathfrak {M}}}_6^{8}=\left( {{\mathfrak {a}}}_{12},{{\mathfrak {A}}}_6^6\right) ^5$	${{\mathfrak {a}}}_{12}{{\mathfrak {b}}}_{20}^2$
	$$t^{12} $$		$${{\mathfrak {M}}}_6^{12}=\left( {{\mathfrak {a}}}_{12},{{\mathfrak {M}}}_6^{8}\right) ^4$$	$${{\mathfrak {a}}}_{12}^4$$
	$t^{16}$		${{\mathfrak {M}}}_6^{16}=\left( {{\mathfrak {a}}}_{12},{{\mathfrak {M}}}_6^{12}\right) ^4$	${{\mathfrak {a}}}_{12}^2{{\mathfrak {b}}}_{20}$
	$t^{24}$		${{\mathfrak {M}}}_6^{24}=\left( {{\mathfrak {a}}}_{12},{{\mathfrak {M}}}_6^{16}\right) ^2$	${{\mathfrak {a}}}_{12}^3$
$\mathbb {Y}_8$	$t^4$	${{\mathfrak {A}}}_8^4$	${{\mathfrak {M}}}_8^4={{\mathfrak {A}}}_8^4$	${{\mathfrak {a}}}_{12}^3{{\mathfrak {b}}}_{20}$
	$t^8$		${{\mathfrak {M}}}_8^{8}=\left( {{\mathfrak {a}}}_{12},{{\mathfrak {A}}}_8^4\right) ^4$	${{\mathfrak {a}}}_{12}{{\mathfrak {b}}}_{20}^2$
	$$t^{12} $$		$${{\mathfrak {M}}}_8^{12}=\left( {{\mathfrak {a}}}_{12},{{\mathfrak {M}}}_8^8\right) ^4$$	$${{\mathfrak {a}}}_{12}^4$$
	$t^{16}$		${{\mathfrak {M}}}_8^{16}=\left( {{\mathfrak {a}}}_{12},{{\mathfrak {M}}}_8^{12}\right) ^4$	${{\mathfrak {a}}}_{12}^2{{\mathfrak {b}}}_{20}$
	$t^{20}$		${{\mathfrak {M}}}_8^{20}=\left( {{\mathfrak {b}}}_{20},{{\mathfrak {A}}}_8^4\right) ^2$	${{\mathfrak {b}}}_{20}^2$

At this stage, one could in principle fix any *G*-orbit (exceptional or generic), divide the matrices by the corresponding invariant form (the invariant form vanishing at those points) in order to obtain zero-homogeneous matrices depending on $\lambda =X/Y$. In this paper, we only consider the case of exceptional orbits. This correspond to dividing the matrices by ${{\mathfrak {a}}}^{d_G}$, ${{\mathfrak {b}}}^3$ or ${{\mathfrak {c}}}^2$, where $d_G=3,4$ and 5 for $\mathbb {T}$, $\mathbb {O}$ and $\mathbb {Y}$, respectively. These then form a minimal generating set (over the invariant , , , respectively—see next Sect. 3.4). We denote this minimal generating set by $\langle \hat{M}^1,\ldots ,\hat{M}^{n^2-1}\rangle$; it generates the *G*-Automorphic Lie Algebra.

##### **Definition 3.4**

By $({{\mathfrak {sl}}}(V)\otimes {\mathbb {k}}(\lambda ))_{{{\mathfrak {z}}}}^{G}$, we denote the *G*-Automorphic Lie Algebra based on ${{\mathfrak {g}}}={{\mathfrak {sl}}}_{}(V)$ with homogeneous coefficients having poles at the *G*-orbit $\Gamma _{{\mathfrak {z}}}$, or, equivalently, at the zeros of ${{\mathfrak {z}}}={{\mathfrak {a}}}$, ${{\mathfrak {b}}}$ or ${{\mathfrak {c}}}$.

##### *Remark 3.1*

(Towards Lax Pairs) Defining a Lax operator *L* $\in ({{\mathfrak {sl}}}(V)\otimes {\mathbb {k}}(\lambda ))_{{{\mathfrak {z}}}}^{G}$ gives us a *G*-invariant (automorphic) Lax operator and therefore a *G*-invariant (automorphic) integrable systems of equations (see [23]).

### Zero-Homogeneous Automorphic Functions

For the $\mathbb {T}\mathbb {O}\mathbb {Y}$-groups, the basic relative invariants ${{\mathfrak {a}}},{{\mathfrak {b}}}$ and ${{\mathfrak {c}}}$ have a relation of the form

$$\begin{aligned} C_{{\mathfrak {z}}}^{{\mathfrak {a}}} {{\mathfrak {a}}}^{d_G} +C_{{\mathfrak {z}}}^{{\mathfrak {b}}} {{\mathfrak {b}}}^3 +C_{{\mathfrak {z}}}^{{\mathfrak {c}}} {{\mathfrak {c}}}^2=0, \quad {{\mathfrak {z}}}={{\mathfrak {a}}},{{\mathfrak {b}}},{{\mathfrak {c}}}. \end{aligned}$$

Dividing this relation by ${{\mathfrak {z}}}^{\nu _{{\mathfrak {z}}}}$, with $\nu _{{\mathfrak {a}}}=d_G$, $\nu _{{\mathfrak {b}}}=3$, $\nu _{{\mathfrak {c}}}=2$, and fixing $C_{{\mathfrak {z}}}^{{\mathfrak {z}}}=1$, we obtain a linear relation between two zero-homogeneous invariants  and . For instance, with ${{\mathfrak {z}}}={{\mathfrak {a}}}$, the relation isThe explicit definition in this case is  and . Or, with ${{\mathfrak {z}}}={{\mathfrak {b}}}$, the relation isThe explicit definition in this case is  and .

A relative invariant ${{\mathfrak {z}}}$ is identified with the orbit of a specific group element $g_{{\mathfrak {z}}}$ of order $\nu _{{\mathfrak {z}}}$, such that $d_{{\mathfrak {z}}}\nu _{{\mathfrak {z}}}=|G|$. For each representation *W* of the group, one defines $\kappa _{{\mathfrak {z}}}={1}\big /{2}\,\mathrm {codim} W^{\langle g_{{\mathfrak {z}}}\rangle }$. In Table 21 (Sect. 6), the numbers $\kappa _{{\mathfrak {a}}},\kappa _{{\mathfrak {b}}}, \kappa _{{\mathfrak {c}}}$ are given for different Lie algebras $W={{\mathfrak {g}}}(V)$.

We use  for the invariant related to the relative invariant with the lowest $\kappa$. If there is equality, for instance if $\kappa _{{\mathfrak {a}}}=\kappa _{{\mathfrak {b}}}$, then in  and , one can interchange the  and the . The fully adorned  is overloaded with indices, and one can replace it by , or one could have simply called it . The reason for the use of the  notation at all is that we later on want to be able to make statements about the Chevalley normal form (see Sect. 5) and their isomorphism.

#### *Remark 3.2*

In the ${{\mathfrak {sl}}}(V)$ case, the relative invariant of the highest degree identifies a lowest $\kappa$ (there could be more than one, see Table 21). In other words, $\kappa _{{\mathfrak {z}}} \le \kappa _{{{\mathfrak {z}}}'}$ if $\deg _{{\mathfrak {z}}} \ge \deg _{{{\mathfrak {z}}}'}$.

## Matrices of Invariants

By constructing a basis of invariant vectors for each irreducible representation (see Tables 14, 15, 16), we prepare ourselves for the next step, the computation of the *matrices of invariants*: we change from the standard basis of an irreducible representation to the basis of invariant vectors. The matrices in the new basis will now have their coefficients in the space of invariants. There are two reasons to make this change of basis.

The first is computational: it is much easier to work with the matrices of invariants, e.g. when computing the structure constants. In the computation of the Chevalley normal form for the Lie algebra, we need to find eigenvalues (see Sect. 5), and this is easier in this new basis. The second reason is that when the algebra is in Chevalley normal form, it will be natural to ask whether the algebra is isomorphic to another case. This *isomorphism question* is difficult to settle, unless one has an explicit way to go from one case to the next. And this is exactly what the matrices of invariants provide. When everything is in Chevalley normal form, the matrices of invariants have been reduced to elementary matrices with invariant coefficients. To analyse them, one can now use permutations and scalings with ${\mathbb {I}}$ and ${\mathbb {J}}$. This limits the problem enough that one can finally answer the isomorphism question.

**Table 14:** Bases of invariant vectors for $\mathbb {T}^\flat$

irrep	Molien	Ground form	Invariant vector	Multiplier
$\mathbb {T}_{2}$	$t^4$	${{\mathfrak {v}}}_{2}^4$	${{\mathfrak {v}}}_{2}^4={{\mathfrak {v}}}_{2}^4$	1
$\mathbb {T}_{3}$	$t^4$	${{\mathfrak {v}}}_{3}^4$	${{\mathfrak {v}}}_{3}^4={{\mathfrak {v}}}_{3}^4$	1
$\mathbb {T}_{4}^\flat$	*t*	${{\mathfrak {v}}}_{4}^1$	${{\mathfrak {v}}}_{4}^1={{\mathfrak {v}}}_{4}^1$	$\bar{{{\mathfrak {a}}}}_{6}$
	$t^7$		${{\mathfrak {v}}}_{4}^7=\left( \bar{{{\mathfrak {b}}}}_{8},{{\mathfrak {v}}}_{4}^1\right) ^1$	1
$\mathbb {T}_{5,6}^\flat$	$t^3$	${{\mathfrak {v}}}_{5,6}^3$	${{\mathfrak {v}}}_{5,6}^3={{\mathfrak {v}}}_{5,6}^3$	$\bar{{{\mathfrak {b}}}}_{8}$
	$t^5$		${{\mathfrak {v}}}_{5,6}^5=\left( \bar{{{\mathfrak {a}}}}_{6},{{\mathfrak {v}}}_{5,6}^3\right) ^2$	$\bar{{{\mathfrak {a}}}}_{6}$
$\mathbb {T}_7$	$t^2$	${{\mathfrak {v}}}_7^2$	${{\mathfrak {v}}}_7^2={{\mathfrak {v}}}_7^2$	$\bar{{{\mathfrak {b}}}}_{8}$
	$t^4$		${{\mathfrak {v}}}_7^4=\left( \bar{{{\mathfrak {a}}}}_{6},{{\mathfrak {v}}}_7^2\right) ^2$	$\bar{{{\mathfrak {a}}}}_{6}$
	$t^{10}$		${{\mathfrak {v}}}_7^{10}=\left( \bar{{{\mathfrak {c}}}}_{12},{{\mathfrak {v}}}_7^2\right) ^2$	1

### *Example 4.1*

In the case of ${{\mathfrak {sl}}}_{2}(\mathbb {T}_5^\flat )$, one has the invariant matrix

$$\begin{aligned} \vartheta ^{{{\mathfrak {sl}}}_{2}(\mathbb {T}_5^\flat )}\left( {{\mathfrak {A}}}_7^2\right) = \begin{pmatrix} XY &{}\quad 1/2Y^2 \\ - 2X^2 &{}\quad - XY \end{pmatrix}. \end{aligned}$$

(cf. Example 3.6). We consider the basis of invariant vectors

$$\begin{aligned} \vartheta ^{\mathbb {T}_5^\flat }\left( {{\mathfrak {v}}}_5^3\right) = \begin{pmatrix} Y^3 + 4X^3 \\ 6XY^2 \end{pmatrix}, \end{aligned}$$

$$\begin{aligned} \vartheta ^{\mathbb {T}_5^\flat }\left( {{\mathfrak {v}}}_5^5\right) = 6635520\begin{pmatrix} -XY^4 - X^4Y \\ 2 X^2Y^3 + 2X^5 \end{pmatrix}. \end{aligned}$$

After making everything zero homogeneous, the matrix of invariants of ${{\mathfrak {M}}}_7^{4}=(\bar{{{\mathfrak {a}}}}_{6},{{\mathfrak {A}}}_7^{2})^2$ becomes


**Table 15:** Bases of invariant vectors for $\mathbb {O}^\flat$

irrep	Molien	Ground form	Invariant vector	Multiplier
$\mathbb {O}_{2}$	$t^6$	${{\mathfrak {v}}}_{2}^6$	${{\mathfrak {v}}}_{2}^6={{\mathfrak {v}}}_{2}^6$	1
$\mathbb {O}_{3}$	$t^4$	${{\mathfrak {v}}}_{3}^4$	${{\mathfrak {v}}}_{3}^4={{\mathfrak {v}}}_{3}^4$	$\bar{{{\mathfrak {b}}}}_{12}$
	$t^8$		${{\mathfrak {v}}}_{3}^8=\left( \bar{{{\mathfrak {a}}}}_{8},{{\mathfrak {v}}}_{3}^4\right) ^2$	$\bar{{{\mathfrak {a}}}}_{8}$
$\mathbb {O}_{4}^\flat$	*t*	${{\mathfrak {v}}}_{4}^1$	${{\mathfrak {v}}}_{4}^1={{\mathfrak {v}}}_{4}^1$	$\bar{{{\mathfrak {a}}}}_{8}^2$
	$t^{17}$		${{\mathfrak {v}}}_{4}^{17}=\left( \bar{{{\mathfrak {c}}}}_{18},{{\mathfrak {v}}}_{4}^1\right) ^1$	1
$\mathbb {O}_{5}^\flat$	$t^5$	${{\mathfrak {v}}}_{5}^5$	${{\mathfrak {v}}}_{5}^{5}={{\mathfrak {v}}}_{5}^5$	$\bar{{{\mathfrak {a}}}}_{8}$
	$t^{13}$		${{\mathfrak {v}}}_{5}^{13}=\left( \bar{{{\mathfrak {b}}}}_{12},{{\mathfrak {v}}}_{5}^5\right) ^2$	1
$\mathbb {O}_6$	$t^4$	${{\mathfrak {v}}}_6^4$	${{\mathfrak {v}}}_6^4={{\mathfrak {v}}}_6^4$	$\bar{{{\mathfrak {a}}}}_{8}^2$
	$t^8$		${{\mathfrak {v}}}_6^8=\left( \bar{{{\mathfrak {a}}}}_{8},{{\mathfrak {v}}}_6^4\right) ^2$	$\bar{{{\mathfrak {b}}}}_{12}$
	$t^{12}$		${{\mathfrak {v}}}_6^{12}=\left( \bar{{{\mathfrak {b}}}}_{12},{{\mathfrak {v}}}_6^4\right) ^2$	$\bar{{{\mathfrak {a}}}}_{8}$
$\mathbb {O}_7$	$t^2$	${{\mathfrak {v}}}_7^2$	${{\mathfrak {v}}}_7^2={{\mathfrak {v}}}_7^2$	$\bar{{{\mathfrak {a}}}}_{8}^2$
	$t^6$		${{\mathfrak {v}}}_7^6=\left( \bar{{{\mathfrak {a}}}}_{8},{{\mathfrak {v}}}_7^2\right) ^2$	$\bar{{{\mathfrak {b}}}}_{12}$
	$t^{10}$		${{\mathfrak {v}}}_7^{10}=\left( \bar{{{\mathfrak {b}}}}_{12},{{\mathfrak {v}}}_7^2\right) ^2$	$\bar{{{\mathfrak {a}}}}_{8}$
$\mathbb {O}_8^\flat$	$t^5$	${{\mathfrak {v}}}_8^3$	${{\mathfrak {v}}}_8^5=\left( \bar{{{\mathfrak {a}}}}_{8},{{\mathfrak {v}}}_8^3\right) ^3$	$\bar{{{\mathfrak {a}}}}_{8}^2$
	$t^9$		${{\mathfrak {v}}}_8^9=\left( \bar{{{\mathfrak {a}}}}_{8},{{\mathfrak {v}}}_8^3\right) ^1$	$\bar{{{\mathfrak {b}}}}_{12}$
	$t^9$		${{\mathfrak {w}}}_8^9=\left( \bar{{{\mathfrak {b}}}}_{12},{{\mathfrak {v}}}_8^3\right) ^3$	$\bar{{{\mathfrak {b}}}}_{12}$
	$t^{13}$		${{\mathfrak {v}}}_8^{13}=\left( \bar{{{\mathfrak {b}}}}_{12},{{\mathfrak {v}}}_8^3\right) ^1$	$\bar{{{\mathfrak {a}}}}_{8}$

**Table 16:** Bases of invariant vectors for $\mathbb {Y}^\flat$

irrep	Molien	Ground form	Invariant vector	Multiplier
$\mathbb {Y}_2^\flat$	$t^{11}$	${{\mathfrak {v}}}_2^1$	${{\mathfrak {v}}}_2^{11}=\big ({{\mathfrak {a}}}_{12},{{\mathfrak {v}}}_2^1\big )^1$	${{\mathfrak {a}}}_{12}^4$
	$t^{19}$		${{\mathfrak {v}}}_2^{19}=\big ({{\mathfrak {b}}}_{20},{{\mathfrak {v}}}_2^1\big )^1$	${{\mathfrak {b}}}_{20}^2$
$\mathbb {Y}_{3}^\flat$	$t^{13}$	${{\mathfrak {v}}}_{3}^7$	${{\mathfrak {v}}}_{3}^{13}=\big ({{\mathfrak {a}}}_{12},{{\mathfrak {v}}}_{3}^7\big )^3$	${{\mathfrak {a}}}_{12}^4$
	$t^{17}$		${{\mathfrak {v}}}_{3}^{17}=\big ({{\mathfrak {b}}}_{20},{{\mathfrak {v}}}_{3}^7\big )^1$	${{\mathfrak {a}}}_{12}^2{{\mathfrak {b}}}_{20}$
$\mathbb {Y}_{4}$	$t^{6}$	${{\mathfrak {v}}}_{4}^6$	${{\mathfrak {v}}}_4^6={{\mathfrak {v}}}_4^6$	${{\mathfrak {b}}}_{20}^2$
	$t^{10}$		${{\mathfrak {v}}}_{4}^{10}=\big ({{\mathfrak {a}}}_{12},{{\mathfrak {v}}}_{4}^6\big )^4$	${{\mathfrak {a}}}_{12}^3$
	$t^{14}$		${{\mathfrak {v}}}_{4}^{14}=\big ({{\mathfrak {a}}}_{12},{{\mathfrak {v}}}_{4}^6\big )^2$	${{\mathfrak {a}}}_{12}{{\mathfrak {b}}}_{20}$
$\mathbb {Y}_{5}$	$t^2$	${{\mathfrak {v}}}_{5}^2$	${{\mathfrak {v}}}_5^2={{\mathfrak {v}}}_{5}^2$	${{\mathfrak {b}}}_{20}^2$
	$t^{10}$		${{\mathfrak {v}}}_{5}^{10}=\big ({{\mathfrak {a}}}_{12},{{\mathfrak {v}}}_{5}^2\big )^2$	${{\mathfrak {a}}}_{12}{{\mathfrak {b}}}_{20}$
	$t^{18}$		${{\mathfrak {v}}}_{5}^{18}=\big ({{\mathfrak {b}}}_{20},{{\mathfrak {v}}}_{5}^2\big )^2$	${{\mathfrak {a}}}_{12}^2$
$\mathbb {Y}_6$	$t^8$	${{\mathfrak {v}}}_6^6$	${{\mathfrak {v}}}_6^8=\big ({{\mathfrak {a}}}_{12},{{\mathfrak {v}}}_6^6\big )^5$	${{\mathfrak {b}}}_{20}^2$
	$t^{12}$		${{\mathfrak {v}}}_6^{12}=\big ({{\mathfrak {a}}}_{12},{{\mathfrak {v}}}_6^6\big )^3$	${{\mathfrak {a}}}_{12}^3$
	$t^{16}$		${{\mathfrak {v}}}_6^{16}=\big ({{\mathfrak {a}}}_{12},{{\mathfrak {v}}}_6^6\big )^1$	${{\mathfrak {a}}}_{12}{{\mathfrak {b}}}_{20}$
	$t^{24}$		${{\mathfrak {v}}}_6^{24}=\big ({{\mathfrak {b}}}_{20},{{\mathfrak {v}}}_6^6\big )^1$	${{\mathfrak {a}}}_{12}^2$
$\mathbb {Y}_7^\flat$	$t^3$	${{\mathfrak {v}}}_7^3$	${{\mathfrak {v}}}_7^3={{\mathfrak {v}}}_7^3$	${{\mathfrak {a}}}_{12}^4$
	$t^{11}$		${{\mathfrak {v}}}_7^{11}=\big ({{\mathfrak {a}}}_{12},{{\mathfrak {v}}}_7^3\big )^2$	${{\mathfrak {b}}}_{20}^2$
	$t^{19}$		${{\mathfrak {v}}}_7^{19}=\big ({{\mathfrak {b}}}_{20},{{\mathfrak {v}}}_7^3\big )^2$	${{\mathfrak {a}}}_{12}{{\mathfrak {b}}}_{20}$
	$t^{27}$		${{\mathfrak {v}}}_7^{27}=\big ({{\mathfrak {c}}}_{30},{{\mathfrak {v}}}_7^3\big )^3$	${{\mathfrak {a}}}_{12}^2$
$\mathbb {Y}_8$	$t^4$	${{\mathfrak {v}}}_8^4$	${{\mathfrak {v}}}_8^4={{\mathfrak {v}}}_8^4$	${{\mathfrak {a}}}_{12}^4$
	$t^8$		${{\mathfrak {v}}}_8^8=\big ({{\mathfrak {a}}}_{12},{{\mathfrak {v}}}_8^4\big )^4$	${{\mathfrak {a}}}_{12}{{\mathfrak {b}}}_{20}$
	$t^{12}$		${{\mathfrak {v}}}_8^{12}=\big ({{\mathfrak {a}}}_{12},{{\mathfrak {v}}}_8^4\big )^2$	${{\mathfrak {b}}}_{20}^2$
	$t^{16}$		${{\mathfrak {v}}}_8^{16}=\big ({{\mathfrak {b}}}_{20},{{\mathfrak {v}}}_8^4\big )^4$	${{\mathfrak {a}}}_{12}^3$
	$t^{20}$		${{\mathfrak {v}}}_8^{20}=\big ({{\mathfrak {b}}}_{20},{{\mathfrak {v}}}_8^4\big )^2$	${{\mathfrak {a}}}_{12}{{\mathfrak {b}}}_{20}$
$\mathbb {Y}_9^\flat$	$t^7$	${{\mathfrak {v}}}_9^5$	${{\mathfrak {v}}}_9^7=\big ({{\mathfrak {a}}}_{12},{{\mathfrak {v}}}_9^5\big )^5$	${{\mathfrak {a}}}_{12}^4$
	$t^{11}$		${{\mathfrak {v}}}_9^{11}=\big ({{\mathfrak {a}}}_{12},{{\mathfrak {v}}}_9^5\big )^3$	${{\mathfrak {a}}}_{12}^2{{\mathfrak {b}}}_{20}$
	$t^{15}$		${{\mathfrak {v}}}_9^{15}=\big ({{\mathfrak {a}}}_{12},{{\mathfrak {v}}}_9^5\big )^1$	${{\mathfrak {b}}}_{20}^2$
	$t^{15}$		${{\mathfrak {w}}}_9^{15}=\big ({{\mathfrak {b}}}_{20},{{\mathfrak {v}}}_9^5\big )^5$	${{\mathfrak {b}}}_{20}^2$
	$t^{19}$		${{\mathfrak {v}}}_9^{19}=\big ({{\mathfrak {b}}}_{20},{{\mathfrak {v}}}_9^5\big )^3$	${{\mathfrak {a}}}_{12}^3$
	$t^{23}$		${{\mathfrak {v}}}_9^{23}=\big ({{\mathfrak {b}}}_{20},{{\mathfrak {v}}}_9^5\big )^1$	${{\mathfrak {a}}}_{12}{{\mathfrak {b}}}_{20}$

In Sects. 3.3.1–3.3.3, we produced the invariant, zero-homogeneous matrices $\hat{M}^1,\ldots ,\hat{M}^{n^2-1}$. We now produce a list of invariant, homogeneous vectors $\hat{v}_1$,...,$\hat{v}_n$, by taking an invariant vector ${{\mathfrak {v}}}$ multiplied by the corresponding invariant multiplier (see Tables 14, 15, 16). The resulting set $\{\hat{v}_i\}$ generates the invariant vectors over the polynomial invariants. If $\mathbb {T}_i^\flat$ is not a representation of $\mathbb {T}$, there are no invariants in $\mathbb {T}_i^\flat \otimes {\mathbb {k}}[X,Y]$ of degree $|\mathbb {T}|$. In this case, one can try as an alternative the lowest degree for which the dimension is the same as the dimension of the irreducible representation. This is listed in Table 14, 15, and 16.

When we compute $\hat{M}^j \hat{v}_{i}$, the result is an invariant vector, that is, a linear combination with invariant coefficients of degree |*G*| of the basic vectors $\hat{v}_1$,...,$\hat{v}_n$. We denote the coefficient of $\hat{v}_{k}$ by $\psi (\hat{M}^j)_{k,i}$ and obtain the following representation of $\hat{M}^j$:

$$\begin{aligned} \hat{M}^j \hat{v}_{i}=\sum _{k=1}^n\, \psi \left( \hat{M}^j\right) _{k,i}\, \hat{v}_{k}. \end{aligned}$$

This defines the matrix $(\psi (\hat{M}^j))_{k,i}$ which is called the *matrix of invariants* corresponding to $\hat{M}^j$, and we extend $\psi$ linearly. We check that the resulting $n^2 -1$ matrices $\psi (\hat{M}^j)$ are linearly independent over ${\mathbb {k}}[{\mathbb {I}}]$. Observe that the matrices $\psi (\hat{M}^j)$ are not themselves invariants under the standard action, as defined in Sect. 2.1. Consider two invariant matrices $\hat{M}$ and $\hat{N}$

$$\begin{aligned} \hat{N}\hat{M}\hat{v}_{i}= & {} \sum _{k}\, \hat{N}\,\psi (\hat{M})_{k,i}\, \hat{v}_{k}=\sum _{k}\psi (\hat{M})_{k,i}\sum _{l}\psi (\hat{N})_{l,k}\, \hat{v}_{l}\\= & {} \sum _{l}\sum _{k}\psi (\hat{N})_{l,k}\,\psi (\hat{M})_{k,i}\, \hat{v}_{l}=\sum _{l} \left( \psi (\hat{N})\psi (\hat{M})\right) _{l,i}\, \hat{v}_{l}. \end{aligned}$$

It follows then that

$$\begin{aligned}{}[\hat{N} , \hat{M}] \hat{v}_{i}=\sum _{l}\left[ \psi (\hat{N}),\psi (\hat{M})\right] _{l,i} \,\hat{v}_{l} \end{aligned}$$

that is,

$$\begin{aligned} \psi \left( [\hat{N} , \hat{M}]\right) =\left[ \psi (\hat{N}) ,\psi (\hat{M})\right] , \end{aligned}$$

in other words, $\psi$ is a Lie algebra homomorphism.

From the computational point of view and in preparation of the next step (namely the computation of Chevalley normal forms), once one has matrices with invariant coefficients, it makes sense to simplify them eliminating as many ${\mathbb {I}}$s as possible by taking linear combinations, while taking care not to change those matrices of invariants with a ${\mathbb {I}}$-independent characteristic polynomial (see the next Sect. 5).

## Chevalley Normal Form for Automorphic Lie Algebras

Even the most detailed Lie algebra books are a bit vague when it comes down to put a concrete Lie algebra into Chevalley normal form over $\mathbb {C}$. In [11], the theory is derived for arbitrary fields, so this is getting closer to our problem. One can imagine how much is written on how to do this over a polynomial ring. In Bourbaki [1], the switch from the general set-up to fields is quickly made in Chapter 1 after Section 3 (even though this is relaxed again at times later on).

The original Lie algebra $${{\mathfrak {sl}}}(V)$$ is of classical type and belongs to an isomorphism class $$A_h $$, with a corresponding $$h\times h$$ Cartan matrix. Following the way the Chevalley normal form is computed over $$\mathbb {C}$$, the first task is to collect *h* commuting semisimple elements from the Lie algebra, the *Cartan subalgebra* or CSA (see e.g. [7, 15]), denoted by $${{\mathfrak {h}}}$$.

### *Remark 5.1*

In a simple Lie algebra over $\mathbb {C}$, a generic element will be semisimple, and one can construct a CSA around it. In the automorphic case, one requires not only semisimplicity but also that the eigenvalues of the matrices in the CSA are in the field extension ${\mathbb {k}}$, thus restricting the choice considerably. In this sense, one could say that Automorphic Lie Algebras are easier to deal with, which is also reflected by the fact that, at least in the ${{\mathfrak {sl}}}(V)$ case, the characteristic equations could always be solved explicitly over ${\mathbb {k}}$. Working over the field extension of the irreducible representations of the group makes it easier to find explicit solutions, even when the degree of the polynomial is five or six. Of course, the computations are made more intricate by the fact that one works not over ${\mathbb {k}}$, but over .

The construction of the CSA ${{\mathfrak {h}}}$ starts with the search of a semisimple element in the Lie algebra of matrices of invariants such that all its eigenvalues are in ${\mathbb {k}}$. Once such a matrix is found, it is tested for semisimplicity. This is done by considering the reduced characteristic polynomial and checking that the matrix itself satisfies it (in the usual theory over $\mathbb {C}$ one looks for an element without degenerate eigenvalues, but this strategy proved not practical in our case). Such an element, once found, can be put in diagonal form. The eigenvalue computation is done by Singular [9]. We call this element $h_1$ and store it in ${{\mathfrak {h}}}$. We then proceed inductively. We find a semisimple element $h_i$ commuting with all the elements in ${{\mathfrak {h}}}$, but ${\mathbb {k}}$-linearly independent of the elements in ${{\mathfrak {h}}}$. We then diagonalise $h_i$ (leaving the other elements in ${{\mathfrak {h}}}$ diagonal). Then, we add $h_i$ to ${{\mathfrak {h}}}$. We stop when we have *h* elements in ${{\mathfrak {h}}}$. By construction, they are all linearly independent and diagonal matrices. Next, one considers a ${\mathbb {k}}$-linear combination of these matrices to insure that their eigenvalues are constants and equal to the one prescribed by the Cartan matrix [2, Plate I] (corresponding to ${{\mathfrak {sl}}}_{n}(\mathbb {C})$ in the classification of simple Lie algebras).

We now give an algorithm to put the elements in ${{\mathfrak {h}}}$ in canonical form in the case of ${{\mathfrak {sl}}}_{n}(\mathbb {C})$. To this end, for every element $h_j$ in ${{\mathfrak {h}}}$ one computes the differences of the subsequent eigenvalues

$$\begin{aligned} \alpha _i(h_j)= \lambda _i^j -\lambda _{i+1}^j,\quad i=1,\ldots ,n-1. \end{aligned}$$

The canonical basis is the set of elements $H_k=\sum _{j=1}^{n-1}c_{j,k} h_j$ satisfying $\alpha _i(H_k)=a_{i,k}$, where $a_{i,k}$ are entries of the Cartan matrix of $A_{n-1}$. Since the matrix $(\alpha _i(h_j))_{i,j}$ is nondegenerate, one can solve $c_{j,k}$, for each fixed *k*, in the equation

$$\begin{aligned} \alpha _i(H_k)=\alpha _i\left( \sum _{j=1}^{n-1}c_{j,k} h_j\right) =\sum _{j=1}^{n-1}\alpha _i(h_j) c_{j,k} =a_{i,k} \end{aligned}$$

and obtain $$H_k$$.

### *Example 5.1*

Consider, as an example, the case $({{\mathfrak {sl}}}_{}({\mathbb {Y}_4})\otimes {\mathbb {k}}(\lambda ))_{{{\mathfrak {a}}}}^{G}$; one finds the elements $h_1=\mathrm {diag}\{-1,1,0\}$ and $h_2=\mathrm {diag}\{1,0,-1\}\in {{\mathfrak {sl}}}_3$. Let *A* be the ${{\mathfrak {sl}}}_3$ Cartan matrix, and let $E_{i,i}$ be the diagonal elementary matrix with 1 in the *i*th position. We would like to have the CSA basis in the standard form $H_1=E_{1,1}-E_{2,2}$ and $H_2=E_{2,2}-E_{3,3}$. We compute

$$\begin{aligned} \alpha (h)=\begin{pmatrix} \alpha _1(h_1) &{}\quad \alpha _1(h_2) \\ \alpha _2(h_1)&{}\quad \alpha _2(h_2)\end{pmatrix}=\begin{pmatrix} -2 &{}\quad 1 \\ 1 &{}\quad 1 \end{pmatrix}. \end{aligned}$$

The matrix *c* is then

$$\begin{aligned} \alpha (h)^{-1} A = -\frac{1}{3} \begin{pmatrix} 1 &{}\quad -1 \\ -1 &{}\quad -2 \end{pmatrix}\begin{pmatrix} 2 &{}\quad -1 \\ -1 &{}\quad 2 \end{pmatrix}=-\frac{1}{3} \begin{pmatrix} 3 &{}\quad -3 \\ 0 &{}\quad -3 \end{pmatrix}=\begin{pmatrix} -1 &{}\quad 1 \\ 0 &{}\quad 1 \end{pmatrix}, \end{aligned}$$

i.e. $H_1=-h_1$ and $H_2=h_1+h_2$. $H_1$ and $H_2$ form a realisation of *A* in the sense of Kac [12].

Let $M_{\alpha _j}$ be a -linear combination of the generators of the ALiA under investigation; one computes them by solving

$$\begin{aligned}{}[H_i, M_{\pm \alpha _j }]=\pm a_{j,i}M_{\pm \alpha _j }. \end{aligned}$$

The $M_{\alpha _j}$ are called weight vectors (of weight $\alpha _j$). Next one computes $[M_{\pm \alpha _j},M_{\pm \alpha _k}]$, $\alpha _j \ne \alpha _k$; if the commutator is not zero, the equation

$$\begin{aligned}{}[H_i, M_{\pm (\alpha _j+\alpha _k) }]=\pm ( a_{j,i}+a_{k,i}) M_{\pm (\alpha _j+\alpha _k)}\, \end{aligned}$$

is solved. Recursively, one computes all the weight vectors in the Chevalley normal form. When all weight vectors have been computed, it is explicitly checked that the transformation from the old generators to this new basis is invertible over .

Notice that we do not have an existence proof of a Chevalley normal form; however, the computation finds always a suitable set of generators such that the algebra is in normal form, so the existence is proven by construction. Since we restrict ourselves to irreducible representations, we only have a finite number of cases to consider.

In the next Sects. 5.2–5.6, we list Chevalley normal forms for $({{\mathfrak {sl}}}(V)\otimes {\mathbb {k}}(\lambda ))_{{{\mathfrak {z}}}}^{G}$, and we prove the following main result:

### **Theorem 5.1**

Let *V* be an irreducible representations of $G^\flat$ and $V^\prime$ be an irreducible representation of $G^{\prime \flat }$, where *G* and $G^\prime$ are isomorphic to the tetrahedral group $\mathbb {T}$, the octahedral group $\mathbb {O}$ or the icosahedral group $\mathbb {Y}$. Let ${{\mathfrak {z}}}$ and ${{\mathfrak {z}}}^\prime$ be *G*, $G^\prime$- classical relative invariants (see Example 3.2); then $({{\mathfrak {g}}}(V)\otimes {\mathbb {k}}(\lambda ))_{{{\mathfrak {z}}}}^{G}$ is isomorphic to $({{\mathfrak {g'}}}(V')\otimes {\mathbb {k}}'(\lambda ))_{{{\mathfrak {z}}}'}^{G'}$ if and only if ${{\mathfrak {g}}}(V)$ is isomorphic to ${{\mathfrak {g}}}^\prime (V^\prime )$ as Lie algebra, where ${{\mathfrak {g}}}, {{\mathfrak {g}}}^\prime ={{\mathfrak {sl}}}$, and $\kappa _{{\mathfrak {z}}}=\kappa _{{{\mathfrak {z}}}'}$, where the $\kappa _{{\mathfrak {z}}}$s can be found in Table 21.

### **Corollary 5.1**

The statement of Theorem 5.1 is true also if one includes the dihedral group $\mathbb {D}_N$ in the list of groups (see [17]).

### Notation

Before proving our result, let us recall the Chevalley normal form of ${{\mathfrak {sl}}}$ over $\mathbb {C}$. It is well known (e.g. [10, Section 25.2]) that the bracket relations of ${{\mathfrak {sl}}}$ over $\mathbb {C}$ can be written in terms of a Cartan–Weyl basis $\langle e_\alpha , e_{\pmb {-}\alpha },h_r\rangle _{\alpha \in \Phi ^+, r=1,\ldots ,\ell }$, where $\Phi ^+$ is a set of positive roots, in which the commutation relations are:

$$\begin{aligned} {[}h_r,h_{s}]= & {} 0\\ {[}h_r, e_\alpha ]= & {} \alpha (h_r)e_\alpha \\ {[} e_\alpha , e_\beta ]= & {} \pm \, e_{\alpha \pmb {+}\beta }, \alpha \pmb {+}\beta \in \Phi \\ {[}e_\alpha ,e_{\pmb {-}\alpha }]= & {} h_\alpha . \end{aligned}$$

Let us also introduce some further notation which will be handy in the following. Consider, as an example, the case $({{\mathfrak {sl}}}_{}({V})\otimes {\mathbb {k}}(\lambda ))_{{{\mathfrak {a}}}}^{G}$, where $V=\mathbb {T}_4^\flat$. After computing the Chevalley normal form as described in the previous section, we findwhere $\alpha _i$ stands for the root. In terms of the original invariant matrices, this Cartan–Weyl basis reads (see also Table 11):We introduce the following short-hand notationwhere we take the sum of all weight vectors; we will refer to this as the *Chevalley model* of the Automorphic Lie Algebra.

#### *Remark 5.2*

$\Vert {{\mathfrak {sl}}}({\mathbb {T}_4^\flat })\Vert$ can be considered as a 1-form with arguments in the root system $A_1$ and values in the space of monomials in  and , the coboundary operator $\mathsf {d}^1$ of which determines the occurrence of these monomials in the structure constants of the ALiA (cf. [18]).

#### *Remark 5.3*

We recall that  is the invariant related to the relative invariant with the lowest $\kappa$, see Sect. 3.4. If there is equality, for instance if $\kappa _{{\mathfrak {a}}}=\kappa _{{\mathfrak {b}}}$, then in  and , one can interchange the  and the , without changing the isomorphism type of the Chevalley normal form.

The Chevalley normal form can be reconstructed from the Cartan matrix (in this case the $1\times 1$ matrix (2)) and from the Chevalley model above. The Lie brackets areFor any $A_h$, given $\alpha =\sum _{k=1}^{h} m_k \alpha _k$ and $m_k\in \mathbb {N}, k=1,\ldots ,h$, the following holds:

$$\begin{aligned}{}[ M_{\alpha } , M_{-\alpha } ]=\langle M_{\alpha } , M_{-\alpha } \rangle H_\alpha , \end{aligned}$$

where $H_\alpha = \sum _{k=1}^{h} m_k H_k$ and $\langle \cdot ,\cdot \rangle$ is the traceform.

The introduced notation suggests how to prove the two necessary conditions for an isomorphism of ALiAs as claimed in Theorem 5.1. First, the base Lie algebras have to be isomorphic. An isomorphism of ALiAs is a $\mathbb {C}[\mathbb {I}]$-linear bijection. Replacing $\mathbb {I}$ by a complex number $\mathbb {I}(\mu )$, we obtain a $\mathbb {C}$-linear bijection between ${{\mathfrak {g}}}(V)^{G_\mu }$ and ${{\mathfrak {g'}}}(V')^{G_\mu }$. For generic points $\mu$, the latter two Lie algebras are the base Lie algebras.

The second necessary condition, namely $\kappa _{{{\mathfrak {z}}}}=\kappa _{{{\mathfrak {z}}}'}$, or equivalently $\{\kappa _{{{\mathfrak {d}}}}\;|\;{{\mathfrak {d}}}\ne {{\mathfrak {z}}}\}=\{\kappa _{{{\mathfrak {d}}}}\;|\;{{\mathfrak {d}}}\ne {{\mathfrak {z}}}'\}$, can be established using the trace form. Indeed, the determinant of the traceform determines the values of $\kappa$ as it is a monomial in $\mathbb {I}$ and $\mathbb {J}$ with powers $2\kappa _{{{\mathfrak {d}}}}$, ${{\mathfrak {d}}}\ne {{\mathfrak {z}}}$. Moreover, this determinant of the traceform is invariant under isomorphisms of ALiAs up to scalars. See [16] for more details.

The harder part of Theorem 5.1 is to show that the given conditions for an isomorphism are also sufficient. We prove this in what follows by listing all cases, ordered by $\dim {{\mathfrak {g}}}(V)$.

#### **Definition 5.1**

We denote by $\Vert A_{n}^{(k,l)}\Vert$ the Automorphic Lie Algebra model based on ${{\mathfrak {sl}}}_{n+1}$ and with *k* s and *l* s in its Cartan–Weyl basis. This defines the ALiA type $A_{n}^{(k,l)}$. It will have the same Cartan matrix as $A_n$ and the specifics of the particular Chevalley model, that is to say, which elements have an  and which have a , will be fixed in the sequel.

Let $\Phi$ be the root system of the base Lie algebra, and let $\Phi ^+$ be a choice of positive roots; together with the model $\Vert A_{n}^{(k,l)}\Vert$, we also considerIn the example above, the sum equals $\mathbb {I}\mathbb {J}$. Computational evidence suggests that this is an invariant.

#### **Definition 5.2**

We denote by $({{\mathfrak {sl}}}_{n}\otimes {\mathbb {k}}(\lambda ))_{{{\mathfrak {z}}}}^{G}$ the *G*-Automorphic Lie Algebra based on ${{\mathfrak {sl}}}(V)$, $\dim (V)=n$, with poles confined at the *G*-orbit $\Gamma _{{\mathfrak {z}}}$, ${{\mathfrak {z}}}={{\mathfrak {a}}}$, ${{\mathfrak {b}}}$ or ${{\mathfrak {c}}}$.

### Automorphic Lie Algebras $({{\mathfrak {sl}}}_{2}\otimes {\mathbb {k}}(\lambda ))_{{{\mathfrak {z}}}}^{G}$

Let the model for $({{\mathfrak {sl}}}_{2}\otimes {\mathbb {k}}(\lambda ))_{{{\mathfrak {z}}}}^{G}$ be

$$\begin{aligned} \Vert A_1^{(1,1)}\Vert =\begin{bmatrix} 0&\quad \mathbb {I} \\ \mathbb {J}&\quad 0 \end{bmatrix},\quad K_{{{\mathfrak {b}}}}({{\mathfrak {sl}}}_{2})_{{\mathfrak {z}}}=\mathbb {I}\mathbb {J} \end{aligned}$$

where $${{\mathfrak {z}}}={{\mathfrak {a}}}$$, $${{\mathfrak {b}}}$$ or $${{\mathfrak {c}}}$$.

#### **Theorem 5.2**

($({{\mathfrak {sl}}}_{2}\otimes {\mathbb {k}}(\lambda ))_{{{\mathfrak {z}}}}^{G}$) All Automorphic Lie Algebras $({{\mathfrak {sl}}}_{2}\otimes {\mathbb {k}}(\lambda ))_{{{\mathfrak {z}}}}^{G}$, ${{\mathfrak {z}}}={{\mathfrak {a}}},{{\mathfrak {b}}},{{\mathfrak {c}}}$, are of type $A_1^{(1,1)}$ and therefore isomorphic.

#### *Proof*

In Tables 17, 18 and 19 we give the Chevalley model together with its intertwining operator $\mathcal{{I}}_{{{\mathfrak {sl}}}(V)}$ with respect to $\Vert A_1^{(1,1)}\Vert$, i.e.

$$\begin{aligned} \Vert {{\mathfrak {sl}}}(V)\Vert \mathcal{{I}}_{{{\mathfrak {sl}}}(V)}=\mathcal{{I}}_{{{\mathfrak {sl}}}(V)} \Vert A_{1}^{(1,1)}\Vert . \end{aligned}$$

$$\square $$

**Table 17:** Chevalley models and intertwining operators for $({{\mathfrak {sl}}}_{2}\otimes {\mathbb {k}}(\lambda ))_{{{\mathfrak {a}}}}^{G}$

Irreducible representation *V*	$\mathbb {T}_{4}$ , $\mathbb {T}_{5}$ , $\mathbb {O}_{3}$ , $\mathbb {O}_{5}$ , $\mathbb {Y}_{2}$ , $\mathbb {Y}_{3}$	$\mathbb {T}_{6}$ , $\mathbb {O}_{4}$
Chevalley model $\Vert {{\mathfrak {sl}}}(V)\Vert$		
Intertwining operator $\mathcal{{I}}_{{{\mathfrak {sl}}}(V)}$		$$\begin{pmatrix} 1&{}\quad 0\\ 0&{}\quad 1\end{pmatrix}$$

**Table 18:** Chevalley models and intertwining operators for $({{\mathfrak {sl}}}_{2}\otimes {\mathbb {k}}(\lambda ))_{{{\mathfrak {b}}}}^{G}$

Irreducible representation *V*	$\mathbb {T}_{4}$ , $\mathbb {T}_{5}$ , $\mathbb {O}_{3}$ , $\mathbb {O}_{5}$	$\mathbb {T}_{6}$ , $\mathbb {Y}_{2}$ , $\mathbb {Y}_{3}$	$\mathbb {O}_{4}$
Chevalley model $\Vert {{\mathfrak {sl}}}(V)\Vert$			
Intertwining operator $\mathcal{{I}}_{{{\mathfrak {sl}}}(V)}$	$$\begin{pmatrix} 1&{}\quad 0\\ 0&{}\quad 1\end{pmatrix}$$

**Table 19:** Chevalley models and intertwining operators for $({{\mathfrak {sl}}}_{2}\otimes {\mathbb {k}}(\lambda ))_{{{\mathfrak {c}}}}^{G}$

Irreducible representation *V*	$\mathbb {T}_{4}$ , $\mathbb {T}_{5}$	$\mathbb {T}_{6}$	$\mathbb {O}_{3}$ , $\mathbb {Y}_{2}$ , $\mathbb {Y}_{3}$	$\mathbb {O}_{4}$, $\mathbb {O}_{5}$
Chevalley model $\Vert {{\mathfrak {sl}}}(V)\Vert$				
Intertwining operator $\mathcal{{I}}_{{{\mathfrak {sl}}}(V)}$				$$\begin{pmatrix} 1&{} 0\\ 0&{}1\end{pmatrix}$$

For the proofs of the following theorems, we refer to Appendix 2.

### Automorphic Lie Algebras $({{\mathfrak {sl}}}_{3}\otimes {\mathbb {k}}(\lambda ))_{{{\mathfrak {z}}}}^{G}$

#### Poles in ${{\mathfrak {a}}}$ and ${{\mathfrak {b}}}$

Let the model for $({{\mathfrak {sl}}}_{3}\otimes {\mathbb {k}}(\lambda ))_{{{\mathfrak {z}}}}^{G}$, ${{\mathfrak {z}}}={{\mathfrak {a}}},{{\mathfrak {b}}}$, be

$$\begin{aligned} \Vert A_2^{(3,2)}\Vert =\begin{bmatrix} 0&\quad \mathbb {I}&\quad \mathbb {I} \\ \mathbb {J}&\quad 0&\quad \mathbb {I} \\ \mathbb {J}&\quad 1&\quad 0 \end{bmatrix},\quad K_{{{\mathfrak {b}}}}({{\mathfrak {sl}}}_{4})_{{{\mathfrak {a}}},{{\mathfrak {b}}}}=\mathbb {I}+2\mathbb {I}\mathbb {J}. \end{aligned}$$

##### **Theorem 5.3**

($({{\mathfrak {sl}}}_{3}\otimes {\mathbb {k}}(\lambda ))_{{{\mathfrak {z}}}}^{G}$, ${{\mathfrak {z}}}={{\mathfrak {a}}},{{\mathfrak {b}}}$) All Automorphic Lie Algebras $({{\mathfrak {sl}}}_{3}\otimes {\mathbb {k}}(\lambda ))_{{{\mathfrak {z}}}}^{G}$, ${{\mathfrak {z}}}={{\mathfrak {a}}},{{\mathfrak {b}}}$, are isomorphic and of type $A_{2}^{(3,2)}$.

#### Poles in ${{\mathfrak {c}}}$

Let the model for $({{\mathfrak {sl}}}_{3}\otimes {\mathbb {k}}(\lambda ))_{{{\mathfrak {c}}}}^{G}$ be

$$\begin{aligned} \Vert A_2^{(3,3)}\Vert =\begin{bmatrix} 0&\quad \mathbb {I}&\quad \mathbb {I} \\ \mathbb {J}&\quad 0&\quad \mathbb {I} \\ \mathbb {J}&\quad \mathbb {J}&\quad 0 \end{bmatrix},\quad K_{{{\mathfrak {b}}}}({{\mathfrak {sl}}}_{4})_{{{\mathfrak {c}}}}=3\mathbb {I}\mathbb {J}. \end{aligned}$$

##### **Theorem 5.4**

($({{\mathfrak {sl}}}_{3}\otimes {\mathbb {k}}(\lambda ))_{{{\mathfrak {c}}}}^{G}$) All Automorphic Lie Algebras $({{\mathfrak {sl}}}_{3}\otimes {\mathbb {k}}(\lambda ))_{{{\mathfrak {c}}}}^{G}$ are isomorphic and of type $A_{2}^{(3,3)}$.

### Automorphic Lie Algebras $({{\mathfrak {sl}}}_{4}\otimes {\mathbb {k}}(\lambda ))_{{{\mathfrak {z}}}}^{G}$

#### Poles in ${{\mathfrak {a}}}$

Let the model for $({{\mathfrak {sl}}}_{4}\otimes {\mathbb {k}}(\lambda ))_{{{\mathfrak {a}}}}^{G}$ be

$$\begin{aligned} \Vert A_3^{(5,4)}\Vert =\begin{bmatrix} 0&\quad \mathbb {I}&\quad \mathbb {I}&\quad \mathbb {I} \\ 1&\quad 0&\quad 1&\quad \mathbb {I} \\ \mathbb {J}&\quad \mathbb {J}&\quad 0&\quad \mathbb {I} \\ \mathbb {J}&\quad \mathbb {J}&\quad 1&\quad 0 \end{bmatrix},\quad K_{{{\mathfrak {b}}}}({{\mathfrak {sl}}}_{4})_{{{\mathfrak {a}}}}=2\mathbb {I}+\mathbb {J}+3\mathbb {I}\mathbb {J}. \end{aligned}$$

##### **Theorem 5.5**

($({{\mathfrak {sl}}}_{4}\otimes {\mathbb {k}}(\lambda ))_{{{\mathfrak {a}}}}^{G}$) All Automorphic Lie Algebras $({{\mathfrak {sl}}}_{4}\otimes {\mathbb {k}}(\lambda ))_{{{\mathfrak {a}}}}^{G}$ are isomorphic and of type $A_{3}^{(5,4)}$.

#### Poles in ${{\mathfrak {b}}}$

Let the model for $({{\mathfrak {sl}}}_{4}\otimes {\mathbb {k}}(\lambda ))_{{{\mathfrak {b}}}}^{G}$ be

$$\begin{aligned} \Vert A_3^{(6,4)}\Vert =\begin{bmatrix} 0&\quad \mathbb {I}&\quad \mathbb {I}&\quad \mathbb {I} \\ 1&\quad 0&\quad \mathbb {I}&\quad \mathbb {I} \\ \mathbb {J}&\quad \mathbb {J}&\quad 0&\quad \mathbb {I} \\ \mathbb {J}&\quad \mathbb {J}&\quad 1&\quad 0 \end{bmatrix},\quad K_{{{\mathfrak {b}}}}({{\mathfrak {sl}}}_{4})_{{{\mathfrak {c}}}}=2\mathbb {I}+4\mathbb {I}\mathbb {J}. \end{aligned}$$

##### **Theorem 5.6**

($({{\mathfrak {sl}}}_{4}\otimes {\mathbb {k}}(\lambda ))_{{{\mathfrak {b}}}}^{G}$) All Automorphic Lie Algebras $({{\mathfrak {sl}}}_{4}\otimes {\mathbb {k}}(\lambda ))_{{{\mathfrak {b}}}}^{G}$ are isomorphic and of type $A_{3}^{(6,4)}$.

#### Poles in ${{\mathfrak {c}}}$

Let the model for $({{\mathfrak {sl}}}_{4}\otimes {\mathbb {k}}(\lambda ))_{{{\mathfrak {c}}}}^{G}$ be

$$\begin{aligned} \Vert A_3^{(6,5)}\Vert =\begin{bmatrix} 0&\quad \mathbb {I}&\quad \mathbb {I}&\quad \mathbb {I} \\ \mathbb {J}&\quad 0&\quad \mathbb {I}&\quad \mathbb {I} \\ \mathbb {J}&\quad \mathbb {J}&\quad 0&\quad \mathbb {I} \\ \mathbb {J}&\quad \mathbb {J}&\quad 1&\quad 0 \end{bmatrix},\quad K_{{{\mathfrak {b}}}}({{\mathfrak {sl}}}_{4})_{{{\mathfrak {c}}}}=\mathbb {I}+5\mathbb {I}\mathbb {J}. \end{aligned}$$

##### **Theorem 5.7**

($({{\mathfrak {sl}}}_{4}\otimes {\mathbb {k}}(\lambda ))_{{{\mathfrak {c}}}}^{G}$) All Automorphic Lie Algebras $({{\mathfrak {sl}}}_{4}\otimes {\mathbb {k}}(\lambda ))_{{{\mathfrak {c}}}}^{G}$ are isomorphic and of type $A_{3}^{(6,5)}$.

### Automorphic Lie Algebras $({{\mathfrak {sl}}}_{5}\otimes {\mathbb {k}}(\lambda ))_{{{\mathfrak {z}}}}^{G}$

#### Poles in ${{\mathfrak {a}}}$

Let the model for $({{\mathfrak {sl}}}_{5}\otimes {\mathbb {k}}(\lambda ))_{{{\mathfrak {a}}}}^{G}$ be

$$\begin{aligned} \Vert A_4^{(8,6)}\Vert =\begin{bmatrix} 0&\quad 1&\quad \mathbb {I}&\quad \mathbb {I}&\quad \mathbb {I} \\ 1&\quad 0&\quad \mathbb {I}&\quad \mathbb {I}&\quad \mathbb {I} \\ \mathbb {J}&\quad \mathbb {J}&\quad 0&\quad 1&\quad \mathbb {I} \\ \mathbb {J}&\quad \mathbb {J}&\quad 1&\quad 0&\quad \mathbb {I} \\ \mathbb {J}&\quad \mathbb {J}&\quad 1&\quad 1&\quad 0 \end{bmatrix},\quad K_{{{\mathfrak {b}}}}({{\mathfrak {sl}}}_{5})_{{{\mathfrak {a}}}}=2+2\mathbb {I}+6\mathbb {I}\mathbb {J}. \end{aligned}$$

##### **Theorem 5.8**

($({{\mathfrak {sl}}}_{5}\otimes {\mathbb {k}}(\lambda ))_{{{\mathfrak {a}}}}^{G}$) All Automorphic Lie Algebras $({{\mathfrak {sl}}}_{5}\otimes {\mathbb {k}}(\lambda ))_{{{\mathfrak {a}}}}^{G}$ are isomorphic and of type $A_{4}^{(8,6)}$.

#### Poles in $\beta$

Let the model for $({{\mathfrak {sl}}}_{5}\otimes {\mathbb {k}}(\lambda ))_{{{\mathfrak {b}}}}^{G}$ be

$$\begin{aligned} \Vert A_4^{(10,6)}\Vert =\begin{bmatrix} 0&\quad \mathbb {I}&\quad \mathbb {I}&\quad \mathbb {I}&\quad \mathbb {I} \\ 1&\quad 0&\quad \mathbb {I}&\quad \mathbb {I}&\quad \mathbb {I} \\ \mathbb {J}&\quad \mathbb {J}&\quad 0&\quad \mathbb {I}&\quad \mathbb {I} \\ \mathbb {J}&\quad \mathbb {J}&\quad 1&\quad 0&\quad \mathbb {I} \\ \mathbb {J}&\quad \mathbb {J}&\quad 1&\quad 1&\quad 0 \end{bmatrix},\quad K_{{{\mathfrak {b}}}}({{\mathfrak {sl}}}_{5})_{{{\mathfrak {b}}}}=4\mathbb {I}+6\mathbb {I}\mathbb {J}. \end{aligned}$$

##### **Theorem 5.9**

($({{\mathfrak {sl}}}_{5}\otimes {\mathbb {k}}(\lambda ))_{{{\mathfrak {b}}}}^{G}$) All Automorphic Lie Algebras $({{\mathfrak {sl}}}_{5}\otimes {\mathbb {k}}(\lambda ))_{{{\mathfrak {b}}}}^{G}$ are isomorphic and of type $A_{4}^{(10,6)}$.

#### Pole in ${{\mathfrak {c}}}$

Let the model for $({{\mathfrak {sl}}}_{5}\otimes {\mathbb {k}}(\lambda ))_{{{\mathfrak {c}}}}^{G}$ be

$$\begin{aligned} \Vert A_4^{(10,8)}\Vert =\begin{bmatrix} 0&\quad \mathbb {I}&\quad \mathbb {I}&\quad \mathbb {I}&\quad \mathbb {I} \\ 1&\quad 0&\quad \mathbb {I}&\quad \mathbb {I}&\quad \mathbb {I} \\ \mathbb {J}&\quad \mathbb {J}&\quad 0&\quad \mathbb {I}&\quad \mathbb {I} \\ \mathbb {J}&\quad \mathbb {J}&\quad \mathbb {J}&\quad 0&\quad \mathbb {I} \\ \mathbb {J}&\quad \mathbb {J}&\quad \mathbb {J}&\quad 1&\quad 0 \end{bmatrix},\quad K_{{{\mathfrak {b}}}}({{\mathfrak {sl}}}_{5})_{{{\mathfrak {c}}}}=2\mathbb {I}+8\mathbb {I}\mathbb {J}. \end{aligned}$$

##### **Theorem 5.10**

($({{\mathfrak {sl}}}_{5}\otimes {\mathbb {k}}(\lambda ))_{{{\mathfrak {c}}}}^{G}$) All Automorphic Lie Algebras $({{\mathfrak {sl}}}_{5}\otimes {\mathbb {k}}(\lambda ))_{{{\mathfrak {c}}}}^{G}$ are isomorphic and of type $A_{4}^{(10,8)}$.

### Automorphic Lie Algebras $({{\mathfrak {sl}}}_{6}\otimes {\mathbb {k}}(\lambda ))_{{{\mathfrak {z}}}}^{G}$

#### Poles in ${{\mathfrak {a}}}$

Let the model for $({{\mathfrak {sl}}}_{6}\otimes {\mathbb {k}}(\lambda ))_{{{\mathfrak {a}}}}^{G}$ be

$$\begin{aligned} \Vert A_5^{(12,9)}\Vert =\begin{bmatrix} 0&\quad 1&\quad \mathbb {I}&\quad \mathbb {I}&\quad \mathbb {I}&\quad \mathbb {I} \\ 1&\quad 0&\quad \mathbb {I}&\quad \mathbb {I}&\quad \mathbb {I}&\quad \mathbb {I} \\ 1&\quad 1&\quad 0&\quad 1&\quad \mathbb {I}&\quad \mathbb {I} \\ \mathbb {J}&\quad \mathbb {J}&\quad \mathbb {J}&\quad 0&\quad \mathbb {I}&\quad \mathbb {I} \\ \mathbb {J}&\quad \mathbb {J}&\quad \mathbb {J}&\quad 1&\quad 0&\quad 1 \\ \mathbb {J}&\quad \mathbb {J}&\quad \mathbb {J}&\quad 1&\quad 1&\quad 0 \end{bmatrix},\quad K_{{{\mathfrak {b}}}}({{\mathfrak {sl}}}_{6})_{{{\mathfrak {a}}}}=2+4\mathbb {I}+\mathbb {J}+8\mathbb {I}\mathbb {J}. \end{aligned}$$

##### **Theorem 5.11**

($({{\mathfrak {sl}}}_{6}\otimes {\mathbb {k}}(\lambda ))_{{{\mathfrak {a}}}}^{G}$) All Automorphic Lie Algebras $({{\mathfrak {sl}}}_{6}\otimes {\mathbb {k}}(\lambda ))_{{{\mathfrak {a}}}}^{G}$ are isomorphic and of type $A_{5}^{(12,9)}$.

#### Poles in ${{\mathfrak {b}}}$

Let the model for $({{\mathfrak {sl}}}_{6}\otimes {\mathbb {k}}(\lambda ))_{{{\mathfrak {b}}}}^{G}$ be

$$\begin{aligned} \Vert A_5^{(14,9)}\Vert =\begin{bmatrix} 0&\quad 1&\quad \mathbb {I}&\quad \mathbb {I}&\quad \mathbb {I}&\quad \mathbb {I} \\ 1&\quad 0&\quad \mathbb {I}&\quad \mathbb {I}&\quad \mathbb {I}&\quad \mathbb {I} \\ 1&\quad 1&\quad 0&\quad \mathbb {I}&\quad \mathbb {I}&\quad \mathbb {I} \\ \mathbb {J}&\quad \mathbb {J}&\quad \mathbb {J}&\quad 0&\quad \mathbb {I}&\quad \mathbb {I} \\ \mathbb {J}&\quad \mathbb {J}&\quad \mathbb {J}&\quad 1&\quad 0&\quad \mathbb {I} \\ \mathbb {J}&\quad \mathbb {J}&\quad \mathbb {J}&\quad 1&\quad 1&\quad 0 \end{bmatrix},\quad K_{{{\mathfrak {b}}}}({{\mathfrak {sl}}}_{6})_{{{\mathfrak {b}}}}=1+5\mathbb {I}+9\mathbb {I}\mathbb {J}. \end{aligned}$$

##### **Theorem 5.12**

($({{\mathfrak {sl}}}_{6}\otimes {\mathbb {k}}(\lambda ))_{{{\mathfrak {b}}}}^{G}$) All Automorphic Lie Algebras $({{\mathfrak {sl}}}_{6}\otimes {\mathbb {k}}(\lambda ))_{{{\mathfrak {b}}}}^{G}$ are isomorphic and of type $A_{5}^{(14,9)}$.

#### Poles in ${{\mathfrak {c}}}$

Let the model for $({{\mathfrak {sl}}}_{6}\otimes {\mathbb {k}}(\lambda ))_{{{\mathfrak {c}}}}^{G}$ be

$$\begin{aligned} \Vert A_5^{(14,12)}\Vert =\begin{bmatrix} 0&\quad 1&\quad \mathbb {I}&\quad \mathbb {I}&\quad \mathbb {I}&\quad \mathbb {I} \\ 1&\quad 0&\quad \mathbb {I}&\quad \mathbb {I}&\quad \mathbb {I}&\quad \mathbb {I} \\ \mathbb {J}&\quad \mathbb {J}&\quad 0&\quad \mathbb {I}&\quad \mathbb {I}&\quad \mathbb {I} \\ \mathbb {J}&\quad \mathbb {J}&\quad 1&\quad 0&\quad \mathbb {I}&\quad \mathbb {I} \\ \mathbb {J}&\quad \mathbb {J}&\quad \mathbb {J}&\quad \mathbb {J}&\quad 0&\quad \mathbb {I} \\ \mathbb {J}&\quad \mathbb {J}&\quad \mathbb {J}&\quad \mathbb {J}&\quad 1&\quad 0 \end{bmatrix},\quad K_{{{\mathfrak {b}}}}({{\mathfrak {sl}}}_{6})_{{{\mathfrak {c}}}}=1+2\mathbb {I}+12\mathbb {I}\mathbb {J}. \end{aligned}$$

##### **Theorem 5.13**

($({{\mathfrak {sl}}}_{6}\otimes {\mathbb {k}}(\lambda ))_{{{\mathfrak {c}}}}^{G}$) All Automorphic Lie Algebras $({{\mathfrak {sl}}}_{6}\otimes {\mathbb {k}}(\lambda ))_{{{\mathfrak {c}}}}^{G}$ are isomorphic and of type $A_{5}^{(14,12)}$.

We have now proved Theorem 5.1 modulo the proofs in Appendix 2.

## Invariants of Automorphic Lie Algebras

In this section, we consider *invariants* of Automorphic Lie Algebras [16]. These are defined as properties of Automorphic Lie Algebras $({{\mathfrak {g}}}(V)\otimes {\mathbb {k}}(\lambda ))_{{{\mathfrak {z}}}}^{G}$ that are independent of the particular reduction group *G* and its representation *V*. That is, properties which only depend on the base Lie algebra and the orbit of poles. The isomorphism question asks whether the Lie algebra structure is an invariant, and this paper affirms this for ${{\mathfrak {g}}}={{\mathfrak {sl}}}$, cf. Theorem 5.1.

We saw already in Sect. 3.3 that the number of generators is an invariant, related to the dimension of the underlying vector space *V*. We will give here two more invariants, namely the number of s and s in the Chevalley model, ${{\mathfrak {z}}},\, {{\mathfrak {z}}}',\, {{\mathfrak {z}}}'' = {{\mathfrak {a}}},\, {{\mathfrak {b}}}$ or ${{\mathfrak {c}}}$.

Let $E_{i,j}$ be the elementary matrix with entry equal to 1 at position (*i*, *j*), and zero elsewhere; since the $H_i$ are by construction of the type $E_{i,i}-E_{i+1,i+1}$, the matrices $M_{\pm \alpha _j }$ will be elementary with coefficients in . We find that the coefficients are always one of four types:  or . We also find that the number of s and s is determined by the dimension of ${{\mathfrak {sl}}}(V)$ and choice of ${{\mathfrak {z}}}$ (see Table 20) and consequently independent of the group.

**Table 20:** Numbers  in the Chevalley model, ${{\mathfrak {z}}}={{\mathfrak {a}}},\,{{\mathfrak {b}}}$ or ${{\mathfrak {c}}}$

$\dim \,{{\mathfrak {sl}}}(V)$	3	8	15	24	35
${{\mathfrak {a}}}$	(1,1)	(3,2)	(5,4)	(8,6)	(12,9)
${{\mathfrak {b}}}$	(1,1)	(3,2)	(6,4)	(10,6)	(14,9)
${{\mathfrak {c}}}$	(1,1)	(3,3)	(6,5)	(10,8)	(14,12)

Computations suggest that the numbers in Table 20 are invariant from the choice of the CSA, from the choice of the group *G* and its irreducible representation *V*. In [16], this is in fact shown to be true for general simple Lie algebras ${{\mathfrak {g}}}(V)$, where *V* is an irreducible *G*-module. Moreover, for all base Lie algebras the numbers can be easily derived with the formulawhere $\langle g_{{{\mathfrak {z}}}'} \rangle$ is a stabiliser subgroup of *G* at a zero of ${{\mathfrak {z}}}'$ [19]. This formula enables us to extend the table counting the automorphic functions in the representations for ALiAs to undiscovered territory. Table 21 is taken from [16], where further details can be found.

**Table 21:** Number of automorphic functions in the Chevalley model: $\kappa _{{{\mathfrak {z}}}'}$, ${{\mathfrak {z}}}'={{\mathfrak {a}}},{{\mathfrak {b}}},{{\mathfrak {c}}}$

${{\mathfrak {g}}}$	${{\mathfrak {sl}}}_2, {{\mathfrak {so}}}_3, {{\mathfrak {sp}}}_2$	${{\mathfrak {so}}}_4$	${{\mathfrak {sl}}}_3$	${{\mathfrak {so}}}_5 ,{{\mathfrak {sp}}}_4$	${{\mathfrak {sl}}}_4$	${{\mathfrak {sp}}}_6$	${{\mathfrak {sl}}}_5$	${{\mathfrak {sl}}}_6$
$$\Phi $$	$$A_1$$	$$A_1\oplus A_1$$	$$A_2$$	$$B_2,C_2$$	$$A_3$$	$$C_3$$	$$A_4$$	$$A_5 $$
$\kappa _{{\mathfrak {a}}}$	1	2	3	4	6	8	10	14
$\kappa _{{\mathfrak {b}}}$	1	2	3	3	5	7	8	12
$\kappa _{{\mathfrak {c}}}$	1	2	2	3	4	6	6	9
$\dim {{\mathfrak {g}}}$	3	6	8	10	15	21	24	35

This table extends Table 20 as follows: the pair in the ${{\mathfrak {z}}}$ row in Table 20 consists of $\kappa _{{{\mathfrak {z}}}'}$ and $\kappa _{{{\mathfrak {z}}}''}$ as found in Table 21, where $\{{{\mathfrak {z}}},{{\mathfrak {z}}}', {{\mathfrak {z}}}''\}=\{{{\mathfrak {a}}},{{\mathfrak {b}}},{{\mathfrak {c}}}\}$. Table 21 provides predictions for the orthogonal and symplectic Lie algebras, which have been verified.

The fact that $\dim {{\mathfrak {g}}}=\sum _{{{\mathfrak {z}}}\in \{{{\mathfrak {a}}},{{\mathfrak {b}}},{{\mathfrak {c}}}\}}{1}\big /{2}\,\mathrm {codim} {{\mathfrak {g}}}(V)^{\langle g_{{\mathfrak {z}}}\rangle }$ is also stated in [26] for the case $G={\mathcal {A}}_5$, the alternating group and attributed to Serre. An algebraic proof is given in [16].

We conclude this section, observing that the polynomials $K_{{{\mathfrak {b}}}}({{\mathfrak {sl}}}_{n})_{{\mathfrak {z}}}$ carry the information from Table 20 and actually add extra information on how the s and s are distributed. Computational evidence suggests that these polynomials are also invariants of the ALiAs.

## Conclusions

The paper addresses the problem of classification for Automorphic Lie Algebras $({{\mathfrak {g}}}\otimes {\mathcal {M}}(\overline{\mathbb {C}}))^G_\Gamma$ where the symmetry group *G* is finite, acts on ${{\mathfrak {g}}}$ by inner automorphisms, and the orbit $\Gamma$ is any of the exceptional *G*-orbits in $\overline{\mathbb {C}}$. It presents a complete classification for the case ${{\mathfrak {sl}}}_n(\mathbb {C})$ and proposes a procedure which can be applied to any semisimple Lie algebra ${{\mathfrak {g}}}$; thus, it is universal. The analysis makes use of notions from classical invariant theory, such as group forms, Molien series and transvectants, and combines the completely classical representation theory of finite groups with the slightly more modern Lie algebra theory over a polynomial ring. It is worth stressing that it is precisely the combination of these two subjects that poses the central questions in this study and makes the subject interesting and worth studying.

The *procedure*, loosely speaking, comprises three steps: the first step consists in identifying the Riemann sphere with the complex projective line $\mathbb {CP}^1$ consisting of quotients ${X}\big /{Y}$ of two complex variables by setting $\lambda ={X}\big /{Y}$ (Sect. 2). Möbius transformations on $\lambda$ then correspond to linear transformations on the vector (*X*, *Y*) by the same matrix. Classical invariant theory is then used to find the *G*-invariant subspaces of $\mathbb {C}[X,Y]$-modules, where $\mathbb {C}[X,Y]$ is the ring of polynomials in *X* and *Y*. Step two consists in localising these ring modules of invariants by a choice of multiplicative set of invariants. This choice corresponds to selecting a *G*-orbit $\Gamma _{{\mathfrak {z}}}$ of poles, or equivalently, selecting a relative invariant ${{\mathfrak {z}}}$ vanishing at those points. The set of elements in the localisation of degree zero, i.e. the set of elements which can be expressed as functions of $\lambda$, generates the ALiA (Sect. 3). Step one and two can be generalised to any Lie algebra ${{\mathfrak {g}}}$, as they rely purely on ${{\mathfrak {g}}}(V)$ being a vector space. Once the algebra is computed, it is transformed in the third step into a Chevalley normal form in the spirit of the standard Cartan–Weyl basis (Sect. 5). This final step relies on the algebraic structure of ${{\mathfrak {g}}}(V)$, and it can be extended to any semisimple Lie algebra ${{\mathfrak {g}}}$.

Through computational means, inspired be the theory of semisimple Lie algebras, we demonstrated the existence of a Chevalley normal form for Automorphic Lie Algebras, generalising this classical notion to the case of Lie algebras over a polynomial ring. Moreover, we show that ALiAs associated with $\mathbb {T}\mathbb {O}\mathbb {Y}$ groups (namely, tetrahedral, octahedral and icosahedral groups) depend on the group through the automorphic functions only; thus, they are group independent as Lie algebras. We prove furthermore that $({{\mathfrak {sl}}}\otimes {\mathcal {M}}(\overline{\mathbb {C}}))^G_{\Gamma _{{{\mathfrak {z}}}}}$ and $({{\mathfrak {sl}}}'\otimes {\mathcal {M}}(\overline{\mathbb {C}}))^{G'}_{\Gamma '_{{{\mathfrak {z}}}'}}$ are isomorphic as Lie algebras if and only if $\kappa _{{\mathfrak {z}}}=\kappa _{{{\mathfrak {z}}}'}$ (Theorem 5.1), and we conjecture a similar result for the cases ${{\mathfrak {so}}}$ and ${{\mathfrak {sp}}}$. This surprising uniformity of ALiAs is not yet completely understood. The study of ALiAs over finite fields could provide information on whether the uniformity is an algebraic or geometric phenomenon.

We also introduce the concept of *matrices of invariants* (see Sect. 4); they describe the (multiplicative) action of invariant matrices on invariant vectors. The description of the invariant matrices in terms of this action yields a much simpler representation of the Lie algebra, reducing the computational cost considerably. We believe that the introduction of matrices of invariants is a fundamental step in the problem of classification of ALiAs.

The Cartan–Weyl basis of the matrices of invariants can be seen as a 1-form, with arguments in $\Phi$, the root system of the original Lie algebra, and taking values in the abelian group of monomials in  and . The structure constants of the ALiA are given by taking the coboundary operator $\mathsf {d}^1$ of this 1-form. This leads to a formulation of the isomorphism problem in terms of the action of $\mathrm {Aut}\left( \Phi \right)$ on the closed 2-forms.

Along with the rise of interest in Darboux transformations with finite reduction groups [20, 27] and applications (e.g. [5]), which suggests wide applications of ALiAs within and beyond integrability theory, this work encourages further study of the structure theory of ALiAs and proposes the notion of *invariants* (Sect. 6), see also [16]. These invariants are polynomials in the coefficients of the computed 1-form that are invariant under $\mathrm {Aut}\left( \Phi \right)$ and the addition of trivial terms. Whether these invariants determine the isomorphism is an open question. From a more general perspective, the success of the structure theory and root system cohomology in the absence of a field promises interesting theoretical developments for Lie algebras over a ring.

The theory of ALiAs gives a natural deformation of classical Lie theory that might be of interest to physics. In particular, it retains the Cartan matrix, thus preserving the finitely generated character of the classical theory.

## Appendix 1: Projective Representations and Double Covering Groups

Let *G* be a finite group, and let $\sigma$ be a faithful projective representation of *G* in $\mathbb {C}^2$, that is, $\sigma$ is a mapping from *G* to $\mathrm {GL}_2(\mathbb {C})$ obeying the following

12 $$\begin{aligned} \sigma (g)\,\sigma (h)=c(g,h)\,\sigma (gh),\quad \forall g,h\in G. \end{aligned}$$

Here $c(g,h)\,:\; G\times G\rightarrow \mathbb {C}^{*}$ in (12) is a nontrivial 2-cocycle over $\mathbb {C}^{*}$, the multiplicative group of $\mathbb {C}$ (see for example [39]), satisfying the cocycle identity

$$\begin{aligned} c(x,y)c(xy,z)=c(y,z)c(x,yz). \end{aligned}$$

It follows from the cocycle condition that $c(1,1)=c(1,z)$ and $c(x,1)=c(1,1)$. So if one defines $\tilde{c}(x,y)=c(x,y)c(1,1)^{-1}$, then $\tilde{c}$ is again a cocycle, but now with $\tilde{c}(x,1)$ and $\tilde{c}(1,x)$ equal to 1. It follows that *c*(*x*, *y*) is a root of unity, the order of which divides the group order. If the cocycle is trivial, one can view the projective representation as a representation.

For each of the Platonic groups $\mathbb {T}, \mathbb {O}$ and $\mathbb {Y}$ consider a projective representation $\sigma$. In order to use GAP to compute generating elements, character tables and Molien functions, we need to replace the projective representation by a representation. The time-honoured method to do this is by constructing the covering group $G^\flat$, which is an extension of the group with its second cohomology group: the sequence

$$\begin{aligned} 0\rightarrow H^2(G,\mathbb {Z}) \rightarrow G^\flat \rightarrow G \rightarrow 0 \end{aligned}$$

is exact. The actual construction runs as follows. One defines (with trivial group action) the group cohomology with values in $\mathbb {Z}$ as follows (written in the usual additive way, followed by multiplication as in the definition of the projective representation):

$$\begin{aligned}&\mathsf {d}^0 a(x)=a-a=0\equiv 1\\&\mathsf {d}^1 b(x,y)=b(xy)-b(x)-b(y)\equiv \frac{b(xy)}{b(x)b(y)}\\&\mathsf {d}^2 c(x,y,z)=c(y,z)-c(xy,z)+c(x,yz)-c(x,y)\equiv \frac{c(y,z)c(x,yz)}{c(xy,z)c(x,y)} \end{aligned}$$

Then, the second cohomology group $H^2(G,\mathbb {Z})$ is defined as the quotient of $\ker \mathsf {d}^2$ over $\mathrm {im\ } \mathsf {d}^1$, which is well defined since $\mathsf {d}^2 \mathsf {d}^1$ maps to unity. We can consider $G^\flat$ as the group generated by the pairs $(r,\rho )$, with $r\in G$ and $\rho \in H^2(G,\mathbb {Z})=\mathbb {Z}/2=\langle \pm 1 \rangle$ [32, 33], with multiplication given by

$$\begin{aligned} (x,\xi )(y,\upsilon )=(xy,\xi \upsilon \tilde{c}(x,y)). \end{aligned}$$

Then, the identity is (*e*, 1), since $\tilde{c}(x,1)$ and $\tilde{c}(1,x)$ are both equal to 1. Let us check associativity (and see what motivated the cocycle identity):

$$\begin{aligned} ((x,\xi )(y,\upsilon ))(z,\zeta )= & {} (xy,\xi \upsilon \tilde{c}(x,y))(z,\zeta )\\= & {} ((xy)z,\xi \upsilon \tilde{c}(x,y)\zeta \tilde{c}(xy,z))\\= & {} (x(yz),\xi \upsilon \zeta \tilde{c}(y,z)\tilde{c}(x,yz))\\= & {} (x,\xi )(yz,\upsilon \zeta \tilde{c}(y,z) )\\= & {} (x,\xi )((y,\upsilon )(z,\zeta )). \end{aligned}$$

One defines the inverse of an element by

$$\begin{aligned} (x,\xi )^{-1}=\left( x^{-1},\xi ^{-1} \tilde{c}(x,x^{-1})^{-1}\right) . \end{aligned}$$

On $G^\flat$ we now define a representation $\sigma ^\flat ((x,\xi ))=\xi c(1,1)^{-1}\sigma (x)$. We have indeed

$$\begin{aligned} \sigma ^\flat ((x,\xi ))\sigma ^\flat ((y,\upsilon ))= & {} c(1,1)^{-2}\xi \upsilon \sigma (x)\sigma (y) \\= & {} c(1,1)^{-2}\xi \upsilon c(x,y)\sigma (xy)\\= & {} \sigma ^\flat ((xy,c(1,1)^{-1}\xi \upsilon c(x,y)))\\= & {} \sigma ^\flat ((xy,\xi \upsilon \tilde{c}(x,y)))= \sigma ^\flat ((x,\xi )(y,\upsilon )). \end{aligned}$$

In practice, one can compute the cocycle the other way around, by considering given $\sigma (r)$ and $\sigma (s)$ as generators of $G^\flat$ and computing the group multiplication table.

### *Remark 7.1*

Suppose there exists a section $s:G\rightarrow G^\flat$. This would imply the existence of an element $\zeta \in C^1(G,\mathbb {Z})$, such that $s(g)=(g,\zeta (g))$. Can we do this so that $s(gh)=s(g)s(h)$? In that case *G* can be viewed as a subgroup of $G^\flat$). This would imply

$$\begin{aligned} s(gh)= & {} (gh,\zeta (gh)) \\ s(g)s(h)= & {} (g,\zeta (g))(h,\zeta (h))=(gh,\zeta (g)\zeta (h)c(g,h)) \end{aligned}$$

But this would in turn imply that $c=\mathsf {d}^1 \zeta$ is a coboundary, where in fact the assumption was that *c* was nontrivial.

## Appendix 2: Chevalley Normal Forms

### **Theorem 9.1**

($({{\mathfrak {sl}}}_{3}\otimes {\mathbb {k}}(\lambda ))_{{{\mathfrak {z}}}}^{G}$, ${{\mathfrak {z}}}={{\mathfrak {a}}},{{\mathfrak {b}}}$) All Automorphic Lie Algebras $({{\mathfrak {sl}}}_{3}\otimes {\mathbb {k}}(\lambda ))_{{{\mathfrak {z}}}}^{G}$, ${{\mathfrak {z}}}={{\mathfrak {a}}},{{\mathfrak {b}}}$, are of type $A_2^{(3,2)}$ and therefore isomorphic.

### *Proof*

We give the Chevalley model together with its intertwining operator $\mathcal{{I}}_{{{\mathfrak {sl}}}(V)}$ with respect to $\Vert A_2^{(3,2)}\Vert$ (see Tables 22, 23), i.e.

$$\begin{aligned} \Vert {{\mathfrak {sl}}}(V)\Vert \mathcal{{I}}_{{{\mathfrak {sl}}}(V)}=\mathcal{{I}}_{{{\mathfrak {sl}}}(V)} \Vert A_{2}^{(3,2)}\Vert . \end{aligned}$$

**Table 22:** Chevalley models and intertwining operators for $({{\mathfrak {sl}}}_{3}\otimes {\mathbb {k}}(\lambda ))_{{{\mathfrak {a}}}}^{G}$

Irreducible representation *V*	$\mathbb {T}_{7}$, $\mathbb {Y}_{5}$	$\mathbb {O}_{6}$	$\mathbb {O}_{7}$	$\mathbb {Y}_{4}$
Chevalley model $\Vert {{\mathfrak {sl}}}(V)\Vert$				
Intertwining operator $\mathcal{{I}}_{{{\mathfrak {sl}}}(V)}$	$$\begin{pmatrix} 0&{}\quad 1&{}\quad 0\\ 1&{}\quad 0&{}\quad 0\\ 0&{}\quad 0&{}\quad 1\end{pmatrix}$$		$$\begin{pmatrix} 0&{}\quad 1&{}\quad 0\\ 0&{}\quad 0&{}\quad 1\\ 1&{}\quad 0&{}\quad 0\end{pmatrix}$$

**Table 23:** Chevalley models and intertwining operators for $({{\mathfrak {sl}}}_{3}\otimes {\mathbb {k}}(\lambda ))_{{{\mathfrak {b}}}}^{G}$

irrep *V*	$\mathbb {T}_{7}$	$\mathbb {O}_{6}$	$\mathbb {O}_{7}$	$\mathbb {Y}_{4}$	$\mathbb {Y}_{5}$
Chevalley model $\Vert {{\mathfrak {sl}}}(V)\Vert$					
Intertwining operator $\mathcal{{I}}_{{{\mathfrak {sl}}}(V)}$	$$\begin{pmatrix} 1&{}\quad 0&{}\quad 0\\ 0&{}\quad 1&{}\quad 0\\ 0&{}\quad 0&{}\quad 1\end{pmatrix}$$	$$\begin{pmatrix} 0&{}\quad 1&{}\quad 0\\ 1&{}\quad 0&{}\quad 0\\ 0&{}\quad 0&{}\quad 1\end{pmatrix}$$			

$$\square $$

### **Theorem 9.2**

($({{\mathfrak {sl}}}_{3}\otimes {\mathbb {k}}(\lambda ))_{{{\mathfrak {c}}}}^{G}$) All Automorphic Lie Algebras $({{\mathfrak {sl}}}_{3}\otimes {\mathbb {k}}(\lambda ))_{{{\mathfrak {c}}}}^{G}$ are of type $A_2^{(3,3)}$ and therefore isomorphic.

### *Proof*

We give the Chevalley model together with its intertwining operator $\mathcal{{I}}_{{{\mathfrak {sl}}}(V)}$ with respect to $\Vert A_2^{(3,3)}\Vert$ (see Table 24), i.e.

$$\begin{aligned} \Vert {{\mathfrak {sl}}}(V)\Vert \mathcal{{I}}_{{{\mathfrak {sl}}}(V)}=\mathcal{{I}}_{{{\mathfrak {sl}}}(V)} \Vert A_{2}^{(3,3)}\Vert . \end{aligned}$$

$$\square $$

**Table 24:** Chevalley models and intertwining operators for $({{\mathfrak {sl}}}_{3}\otimes {\mathbb {k}}(\lambda ))_{{{\mathfrak {c}}}}^{G}$

irrep *V*	$\mathbb {T}_{7}$	$\mathbb {O}_{6}$, $\mathbb {Y}_{5}$	$\mathbb {O}_{7}$	$\mathbb {Y}_{4}$
Chevalley model $\Vert {{\mathfrak {sl}}}(V)\Vert$				
Intertwining operator $\mathcal{{I}}_{{{\mathfrak {sl}}}(V)}$				$$\begin{pmatrix} 1&{}\quad 0&{}\quad 0\\ 0&{}\quad 0&{}\quad 1\\ 0&{}\quad 1&{}\quad 0\end{pmatrix}$$

**Table 25:** Chevalley models and intertwining operators for $({{\mathfrak {sl}}}_{4}\otimes {\mathbb {k}}(\lambda ))_{{{\mathfrak {a}}}}^{G}$

irrep *V*	$\mathbb {O}_{8}$	$\mathbb {Y}_{6}$	$\mathbb {Y}_{7}$
Chevalley model $\Vert {{\mathfrak {sl}}}(V)\Vert$			
Intertwining operator $\mathcal{{I}}_{{{\mathfrak {sl}}}(V)}$			

### **Theorem 9.3**

($({{\mathfrak {sl}}}_{4}\otimes {\mathbb {k}}(\lambda ))_{{{\mathfrak {a}}}}^{G}$) All Automorphic Lie Algebras $({{\mathfrak {sl}}}_{4}\otimes {\mathbb {k}}(\lambda ))_{{{\mathfrak {a}}}}^{G}$ are of type $A_3^{(5,4)}$ and therefore isomorphic.

### *Proof*

We give the Chevalley model together with its intertwining operator $\mathcal{{I}}_{{{\mathfrak {sl}}}(V)}$ with respect to $\Vert A_3^{(5,4)}\Vert$ (see Table 25), i.e.

$$\begin{aligned} \Vert {{\mathfrak {sl}}}(V)\Vert \mathcal{{I}}_{{{\mathfrak {sl}}}(V)}=\mathcal{{I}}_{{{\mathfrak {sl}}}(V)} \Vert A_{3}^{(5,4)}\Vert . \end{aligned}$$

### **Theorem 9.4**

($({{\mathfrak {sl}}}_{4}\otimes {\mathbb {k}}(\lambda ))_{{{\mathfrak {b}}}}^{G}$) All Automorphic Lie Algebras $({{\mathfrak {sl}}}_{4}\otimes {\mathbb {k}}(\lambda ))_{{{\mathfrak {b}}}}^{G}$ are of type $A_3^{(6,4)}$ and therefore isomorphic.

### *Proof*

We give the Chevalley model together with its intertwining operator $\mathcal{{I}}_{{{\mathfrak {sl}}}(V)}$ with respect to $\Vert A_3^{(6,4)}\Vert$ (see Table 26), i.e.

$$\begin{aligned} \Vert {{\mathfrak {sl}}}(V)\Vert \mathcal{{I}}_{{{\mathfrak {sl}}}(V)}=\mathcal{{I}}_{{{\mathfrak {sl}}}(V)} \Vert A_{3}^{(6,4)}\Vert . \end{aligned}$$

$$\square $$

### **Theorem 9.5**

($({{\mathfrak {sl}}}_{4}\otimes {\mathbb {k}}(\lambda ))_{{{\mathfrak {c}}}}^{G}$) All Automorphic Lie Algebras $({{\mathfrak {sl}}}_{4}\otimes {\mathbb {k}}(\lambda ))_{{{\mathfrak {c}}}}^{G}$ are of type $A_3^{(6,5)}$ and therefore isomorphic.

### *Proof*

We give the Chevalley model together with its intertwining operator $\mathcal{{I}}_{{{\mathfrak {sl}}}(V)}$ with respect to $\Vert A_3^{(6,5)}\Vert$ (see Table 27), i.e.

$$\begin{aligned} \Vert {{\mathfrak {sl}}}(V)\Vert \mathcal{{I}}_{{{\mathfrak {sl}}}(V)}=\mathcal{{I}}_{{{\mathfrak {sl}}}(V)} \Vert A_{3}^{(6,5)}\Vert . \end{aligned}$$

$$\square $$

**Table 26:** Chevalley models and intertwining operators for $({{\mathfrak {sl}}}_{4}\otimes {\mathbb {k}}(\lambda ))_{{{\mathfrak {b}}}}^{G}$

Irreducible representation *V*	$\mathbb {O}_{8}$	$\mathbb {Y}_{6}$	$\mathbb {Y}_{7}$
Chevalley model $\Vert {{\mathfrak {sl}}}(V)\Vert$			
Intertwining operator $$\mathcal{{I}}_{{{\mathfrak {sl}}}(V)}$$

**Table 27:** Chevalley models and intertwining operators for $({{\mathfrak {sl}}}_{4}\otimes {\mathbb {k}}(\lambda ))_{{{\mathfrak {c}}}}^{G}$

Irreducible representation *V*	$\mathbb {O}_{8}$	$\mathbb {Y}_{6}$	$\mathbb {Y}_{7}$
Chevalley model $\Vert {{\mathfrak {sl}}}(V)\Vert$			
Intertwining operator $\mathcal{{I}}_{{{\mathfrak {sl}}}(V)}$	$$\begin{pmatrix} 1&{}\quad 0&{}\quad 0&{}\quad 0\\ 0&{}\quad 0&{}\quad 0&{}\quad 1\\ 0&{}\quad 1&{}\quad 0&{}\quad 0\\ 0&{}\quad 0&{}\quad 1&{}\quad 0\end{pmatrix}$$	$$\begin{pmatrix} 1&{}\quad 0&{}\quad 0&{}\quad 0\\ 0&{}\quad 1&{}\quad 0&{}\quad 0\\ 0&{}\quad 0&{}\quad 0&{}\quad 1\\ 0&{}\quad 0&{}\quad 1&{}\quad 0\end{pmatrix}$$	$$\begin{pmatrix} 0&{}\quad 0&{}\quad 1&{}\quad 0\\ 1&{}\quad 0&{}\quad 0&{}\quad 0\\ 0&{}\quad 1&{}\quad 0&{}\quad 0\\ 0&{}\quad 0&{}\quad 0&{}\quad 1\end{pmatrix}$$

**Table 28:** $V=\mathbb {Y}_{8}$; Chevalley models and intertwining operators for $({{\mathfrak {sl}}}_{5}\otimes {\mathbb {k}}(\lambda ))_{{{\mathfrak {z}}}}^{G}$, ${{\mathfrak {z}}}={{\mathfrak {a}}},{{\mathfrak {b}}},{{\mathfrak {c}}}$

Poles at $\Gamma _{{{\mathfrak {z}}}}$	$\Gamma _{{{\mathfrak {a}}}}$	$\Gamma _{{{\mathfrak {b}}}}$	$\Gamma _{{{\mathfrak {c}}}}$
Chevalley model $\Vert {{\mathfrak {sl}}}(V)\Vert$			
Intertwining operator $\mathcal{{I}}_{{{\mathfrak {sl}}}(V)}$	$$\begin{pmatrix} 0&{}\quad 0&{}\quad 0&{}\quad 0&{}\quad 1\\ 1&{}\quad 0&{}\quad 0&{}\quad 0&{}\quad 0\\ 0&{}\quad 1&{}\quad 0&{}\quad 0&{}\quad 0\\ 0&{}\quad 0&{}\quad 1&{}\quad 0&{}\quad 0\\ 0&{}\quad 0&{}\quad 0&{}\quad 1&{}\quad 0\end{pmatrix}$$

**Table 29:** $V=\mathbb {Y}_{9}$; Chevalley models and intertwining operators for $({{\mathfrak {sl}}}_{6}\otimes {\mathbb {k}}(\lambda ))_{{{\mathfrak {z}}}}^{G}$, ${{\mathfrak {z}}}={{\mathfrak {a}}},{{\mathfrak {b}}},{{\mathfrak {c}}}$

Poles at $\Gamma _{{{\mathfrak {z}}}}$	$\Gamma _{{{\mathfrak {a}}}}$	$\Gamma _{{{\mathfrak {b}}}}$	$\Gamma _{{{\mathfrak {c}}}}$
Chevalley model $\Vert {{\mathfrak {sl}}}(V)\Vert$			
Inter operator $\mathcal{{I}}_{{{\mathfrak {sl}}}(V)}$	$$\begin{pmatrix} 1&{}\quad 0&{}\quad 0&{}\quad 0&{}\quad 0&{}\quad 0\\ 0&{}\quad 0&{}\quad 0&{}\quad 0&{}\quad 1&{}\quad 0\\ 0&{}\quad 0&{}\quad 0&{}\quad 1&{}\quad 0&{}\quad 0\\ 0&{}\quad 0&{}\quad 1&{}\quad 0&{}\quad 0&{}\quad 0\\ 0&{}\quad 0&{}\quad 0&{}\quad 0&{}\quad 0&{}\quad 1\\ 0&{}\quad 1&{}\quad 0&{}\quad 0&{}\quad 0&{}\quad 0\end{pmatrix}$$	$$\begin{pmatrix} 0&{}\quad 0&{}\quad 0&{}\quad 0&{}\quad 1&{}\quad 0\\ 0&{}\quad 0&{}\quad 0&{}\quad 0&{}\quad 0&{}\quad 1\\ 1&{}\quad 0&{}\quad 0&{}\quad 0&{}\quad 0&{}\quad 0\\ 0&{}\quad 0&{}\quad 0&{}\quad 1&{}\quad 0&{}\quad 0\\ 0&{}\quad 0&{}\quad 1&{}\quad 0&{}\quad 0&{}\quad 0\\ 0&{}\quad 1&{}\quad 0&{}\quad 0&{}\quad 0&{}\quad 0\end{pmatrix}$$	$$\begin{pmatrix} 0&{}\quad 0&{}\quad 0&{}\quad 1&{}\quad 0&{}\quad 0\\ 0&{}\quad 0&{}\quad 1&{}\quad 0&{}\quad 0&{}\quad 0\\ 1&{}\quad 0&{}\quad 0&{}\quad 0&{}\quad 0&{}\quad 0\\ 0&{}\quad 0&{}\quad 0&{}\quad 0&{}\quad 1&{}\quad 0\\ 0&{}\quad 0&{}\quad 0&{}\quad 0&{}\quad 0&{}\quad 1\\ 0&{}\quad 1&{}\quad 0&{}\quad 0&{}\quad 0&{}\quad 0\end{pmatrix}$$

### **Theorem 9.6**

($({{\mathfrak {sl}}}_{5}\otimes {\mathbb {k}}(\lambda ))_{{{\mathfrak {a}}}}^{G}$) All Automorphic Lie Algebras $({{\mathfrak {sl}}}_{5}\otimes {\mathbb {k}}(\lambda ))_{{{\mathfrak {a}}}}^{G}$ are of type $A_4^{(8,6)}$ and therefore isomorphic.

### *Proof*

We give the Chevalley model together with its intertwining operator $\mathcal{{I}}_{{{\mathfrak {sl}}}(V)}$ with respect to $\Vert A_4^{(8,6)}\Vert$ (see Table 28), i.e.

$$\begin{aligned} \Vert {{\mathfrak {sl}}}(V)\Vert \mathcal{{I}}_{{{\mathfrak {sl}}}(V)}=\mathcal{{I}}_{{{\mathfrak {sl}}}(V)} \Vert A_{4}^{(8,6)}\Vert . \end{aligned}$$

$$\square $$

### **Theorem 9.7**

($({{\mathfrak {sl}}}_{5}\otimes {\mathbb {k}}(\lambda ))_{{{\mathfrak {b}}}}^{G}$) All Automorphic Lie Algebras $({{\mathfrak {sl}}}_{5}\otimes {\mathbb {k}}(\lambda ))_{{{\mathfrak {b}}}}^{G}$ are of type $A_4^{(10,6)}$ and therefore isomorphic.

### *Proof*

We give the Chevalley model together with its intertwining operator $\mathcal{{I}}_{{{\mathfrak {sl}}}(V)}$ with respect to $\Vert A_4^{(10,6)}\Vert$ (see Table 28), i.e.

$$\begin{aligned} \Vert {{\mathfrak {sl}}}(V)\Vert \mathcal{{I}}_{{{\mathfrak {sl}}}(V)}=\mathcal{{I}}_{{{\mathfrak {sl}}}(V)} \Vert A_{4}^{(10,6)}\Vert . \end{aligned}$$

$$\square $$

### **Theorem 9.8**

($({{\mathfrak {sl}}}_{4}\otimes {\mathbb {k}}(\lambda ))_{{{\mathfrak {c}}}}^{G}$) All Automorphic Lie Algebras $({{\mathfrak {sl}}}_{5}\otimes {\mathbb {k}}(\lambda ))_{{{\mathfrak {c}}}}^{G}$ are of type $A_4^{(10,8)}$ and therefore isomorphic.

### *Proof*

We give the Chevalley model together with its intertwining operator $\mathcal{{I}}_{{{\mathfrak {sl}}}(V)}$ with respect to $\Vert A_4^{(10,8)}\Vert$ (see Table 28), i.e.

$$\begin{aligned} \Vert {{\mathfrak {sl}}}(V)\Vert \mathcal{{I}}_{{{\mathfrak {sl}}}(V)}=\mathcal{{I}}_{{{\mathfrak {sl}}}(V)} \Vert A_{4}^{(10,8)}\Vert . \end{aligned}$$

$$\square $$

### **Theorem 9.9**

($({{\mathfrak {sl}}}_{6}\otimes {\mathbb {k}}(\lambda ))_{{{\mathfrak {a}}}}^{G}$) All Automorphic Lie Algebras $({{\mathfrak {sl}}}_{6}\otimes {\mathbb {k}}(\lambda ))_{{{\mathfrak {a}}}}^{G}$ are of type $A_5^{(12,9)}$ and therefore isomorphic.

### *Proof*

We give the Chevalley model together with its intertwining operator $\mathcal{{I}}_{{{\mathfrak {sl}}}(V)}$ with respect to $\Vert A_5^{(12,9)}\Vert$ (see Table 29), i.e.

$$\begin{aligned} \Vert {{\mathfrak {sl}}}(V)\Vert \mathcal{{I}}_{{{\mathfrak {sl}}}(V)}=\mathcal{{I}}_{{{\mathfrak {sl}}}(V)} \Vert A_{5}^{(12,9)}\Vert . \end{aligned}$$

$$\square $$

### **Theorem 9.10**

($({{\mathfrak {sl}}}_{6}\otimes {\mathbb {k}}(\lambda ))_{{{\mathfrak {b}}}}^{G}$) All Automorphic Lie Algebras $({{\mathfrak {sl}}}_{6}\otimes {\mathbb {k}}(\lambda ))_{{{\mathfrak {b}}}}^{G}$ are of type $A_5^{(14,9)}$ and therefore isomorphic.

### *Proof*

We give the Chevalley model together with its intertwining operator $\mathcal{{I}}_{{{\mathfrak {sl}}}(V)}$ with respect to $\Vert A_6^{(14,9)}\Vert$ (see Table 29), i.e.

$$\begin{aligned} \Vert {{\mathfrak {sl}}}(V)\Vert \mathcal{{I}}_{{{\mathfrak {sl}}}(V)}=\mathcal{{I}}_{{{\mathfrak {sl}}}(V)} \Vert A_{5}^{(14,9)}\Vert . \end{aligned}$$

$$\square $$

### **Theorem 9.11**

($({{\mathfrak {sl}}}_{6}\otimes {\mathbb {k}}(\lambda ))_{{{\mathfrak {c}}}}^{G}$) All Automorphic Lie Algebras $({{\mathfrak {sl}}}_{6}\otimes {\mathbb {k}}(\lambda ))_{{{\mathfrak {c}}}}^{G}$ are of type $A_5^{(14,12)}$ and therefore isomorphic.

### *Proof*

We give the Chevalley model together with its intertwining operator $\mathcal{{I}}_{{{\mathfrak {sl}}}(V)}$ with respect to $\Vert A_6^{(14,12)}\Vert$ (see Table 29), i.e.

$$\begin{aligned} \Vert {{\mathfrak {sl}}}(V)\Vert \mathcal{{I}}_{{{\mathfrak {sl}}}(V)}=\mathcal{{I}}_{{{\mathfrak {sl}}}(V)} \Vert A_{5}^{(14,12)}\Vert . \end{aligned}$$

$$\square $$
